# New Monoterpenoid Glycosides from the Fruits of *Hypericum patulum* Thunb.

**DOI:** 10.3390/molecules29133075

**Published:** 2024-06-27

**Authors:** Li Jiang, Xue Ma, Yang Wang, Jian-Ping Yang, Yong Huang, Chun-Hua Liu, Yong-Jun Li

**Affiliations:** 1 Engineering Research Center for the Development and Application of Ethnic Medicine and TCM, Ministry of Education/Guizhou Provincial Engineering Research Center for the Development and Application of Ethnic Medicine and TCM, Guizhou Medical University, Guiyang 550004, China; jiangli0324@gmc.edu.cn (L.J.); xuema0111@163.com (X.M.); 2 School of Pharmacy, Guizhou Medical University, Guiyang 550004, China; 15180803607@163.com (Y.W.); yangjianping762@gmail.com (J.-P.Y.); huangy2020@126.com (Y.H.); 3 School of Basic Medical Sciences, Guizhou Medical University, Guiyang 550004, China; 4 Guizhou Provincial Key Laboratory of Pharmaceutics/State Key Laboratory of Functions and Applications of Medicinal Plants, Guizhou Medical University, Guiyang 550004, China; 5 National Engineering Research Center of Miao’s Medicines, Guizhou Medical University, Guiyang 550004, China

**Keywords:** *Hypericum patulum* Thunb., chemical constituents, monoterpenoid glycosides, structure identification

## Abstract

The whole *Hypericum patulum* Thunb. plant is utilized in traditional medicine for its properties of clearing heat, detoxifying, soothing meridians, relaxing the liver, and stopping bleeding. In folk medicine, it is frequently used to treat hepatitis, colds, tonsillitis, and bruises. Phytochemical investigation of a 30% ethanol extract of the fresh ripe fruits of *H. patulum* has resulted in the isolation of two new pinane-type monoterpenoid glycosides **1**–**2**, named patulumside E-F, and three new chain-shaped monoterpenoid glycosides **3**–**5**, named patulumside G-H, J. Their structures were determined using extensive spectroscopic techniques, such as HR-ESI-MS, 1D and 2D NMR spectroscopy, and electronic circular dichroism (ECD) calculation. The anti-inflammatory activities of these compounds were evaluated in the LPS-induced RAW264.7 cells. This research represents the inaugural comprehensive phytochemical study of *H. patulum*, paving the way for further exploration of monoterpenoid glycosides.

## 1. Introduction

The *Hypericum* genus belongs to the Guttiferae family and is highly diverse, accounting for over 80% of the Guttiferae species. There are more than 500 species worldwide, widely distributed across Asia, Europe, North Africa, and North America [1]. In China, there are approximately 55 species and eight subspecies, mainly distributed in East China, South China, Central China, southern Shaanxi, southern Gansu, and northeastern Sichuan [2,3]. The plants have been utilized for treatment of burns, bruising, swelling, inflammation, anxiety, as well as bacterial and viral infections [4,5]. *H. patulum* is extensively utilized in numerous traditional Chinese medicine prescriptions, serving as a vital component in various formulations. Particularly, the fresh fruits of *H. patulum* are used exclusively in a medicinal preparation that blends the traditional pottery techniques of the Miao ethnic group with modern technology to create Pingzhi capsules. These capsules are highly effective in alleviating mild bleeding from internal hemorrhoids and reducing swelling and pain from external hemorrhoids caused by damp-heat accumulation in the large intestine [6]. Up to now, numerous constituents including naphthodianthrones [7], phloroglucinol derivatives [8,9], terpenoids [10], flavonoids [11], and xanthones [12] with documented biological activities have been isolated from *Hypericum patulum* Thunb. However, previous research on *H. patulum* has primarily examined the chemical composition of its flowers, leaves, and aerial parts, with a particular focus on phloroglucinol derivatives [13,14,15]. The chemical constituents of the fresh ripe fruits of *H. patulum.* have not been reported before our research. 

Glycoside compounds are widely present in natural plants and are an important active ingredient. Various plant glycosides, such as flavonoid glycosides, saponin glycosides, and terpenoid glycosides, have been reported to have beneficial effects on conditions such as diabetic nephropathy, neuropathy, retinopathy, and cardiomyopathy [16]. Moreover, plant glycosides, especially laurosides, have shown potential anti-COVID-19 activity [17]. So far, biphenyl ether glycosides, benzophenone glycosides, acylphloroglucinol glycosides, and flavonoid glycosides have been isolated from the *Hypericum* genus. These compounds exhibit neurotrophic activity, myocardial protective effects, antioxidant properties, and anti-Helicobacter pylori activity [18,19,20,21]. We have conducted continuous investigations of the phytochemical variety of the 30% ethanol extract of the fresh ripe fruits of *H. patulum*. In this study, we have reported for the first time the isolation and elucidation of another five new monoterpenoid glycosides (**1**–**5**). Herein, we describe the isolation, structural elucidation, and anti-inflammatory activities of these new compounds.

## 2. Results and Discussion

### 2.1. Structure Determination

**Figure 1:**
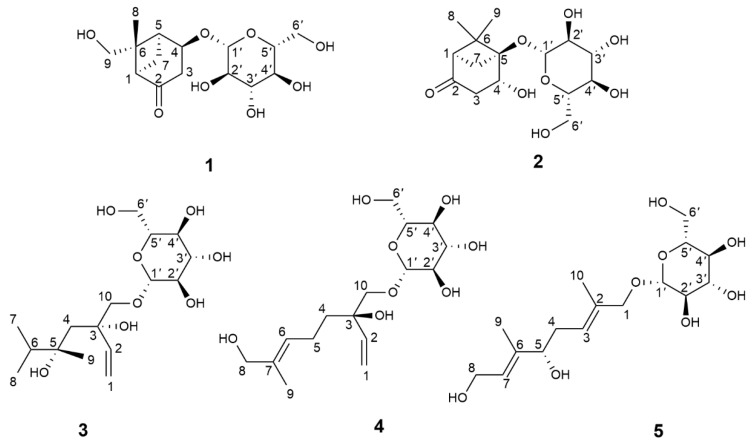
Structures of compounds **1**–**5**.

The 30% ethanol extract of the fresh ripe fruits of *H. patulum* was subjected to repeated column chromatography (CC) using silica gel, Sephadex LH-20, Toyopearl HW-40F, and ODS, resulting in the isolation of five new monoterpenoid glycosides: patulumside E (**1**), patulumside F (**2**), patulumside G (**3**), patulumside H (**4**), and patulumside J (**5**) (structures are shown in Figure 1).

### 2.2. Characterization of Compound ***1***

**Figure 2:**
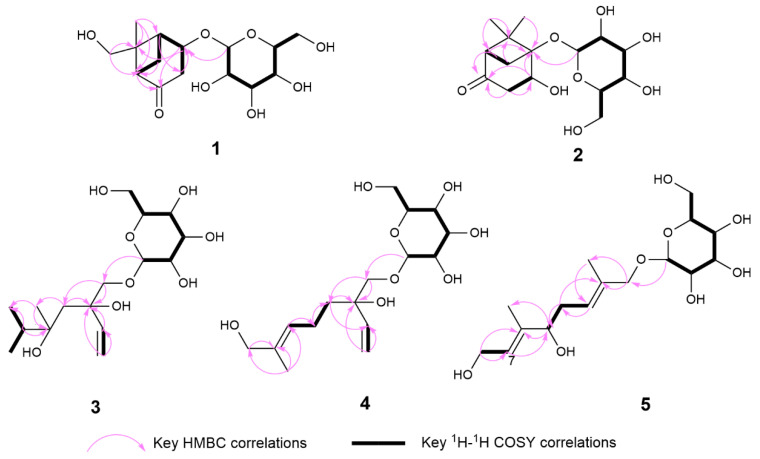
Key ^1^H-^1^H COSY and HMBC correlations of compounds **1**–**5**.

**Figure 3:**
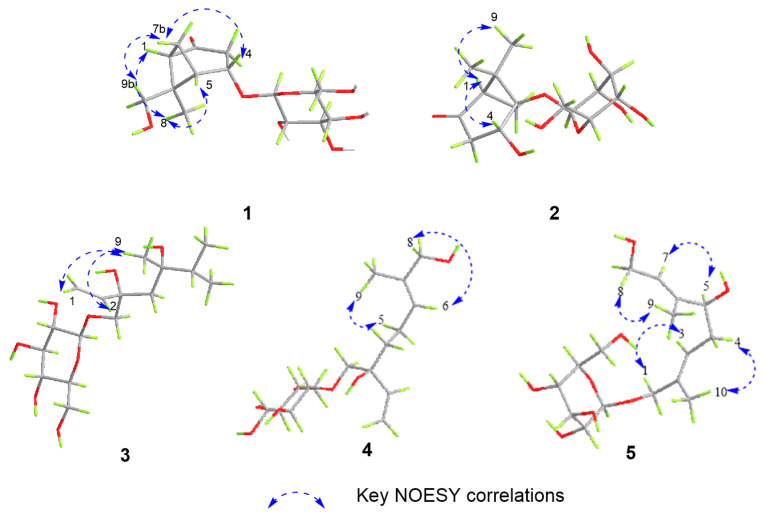
Key NOESY correlations of compounds **1**–**5**.

**Figure 4:**
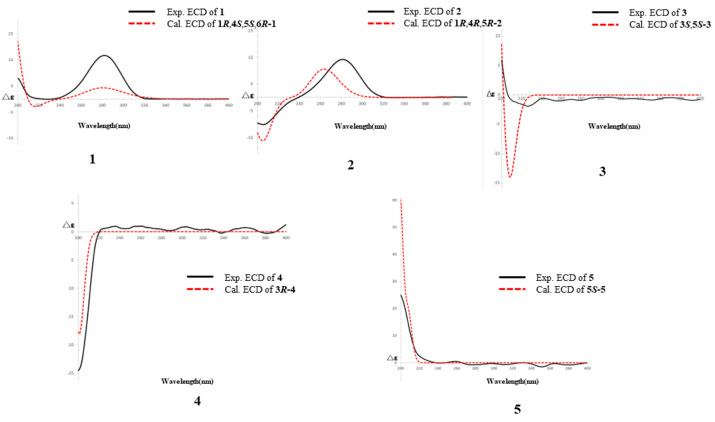
Calculated and experimental ECD spectra of compounds **1**–**5**.

Compound **1** was isolated as a colorless solid, and its molecular formula was determined to be C_15_H_24_O_8_ by HR-ESI-MS (*m*/*z*: 331.1399 [M−H]^−^, calcd. 331.1387; 355.1256 [M+Na]^+^, calcd. 355.1263). The ^1^H-NMR spectrum showed the presence of a methyl signal at *δ*_H_ 1.44 (3H, s, H_3_-8); two methylene signals at *δ*_H_ 3.19 (1H, overlapped, H-3a), 2.63 (1H, m, H-7a), 2.52 (1H, dd, *J* = 20.0, 1.7 Hz, H-3b), and 1.51 (1H, d, *J* = 10.9 Hz, H-7b); a hydroxymethyl signal at *δ*_H_ 3.67 (1H, d, *J* = 11.4 Hz, H-9a) and 3.13 (1H, d, *J* = 11.4 Hz, H-9b); three methine signals at *δ*_H_ 4.64 (1H, m, H-4), 2.70 (1H, m, H-5), and 2.56 (1H t, *J* = 5.0 Hz, H-1); and one anomeric proton signal at *δ*_H_ 4.43 (1H, d, *J* = 7.7 Hz, H-1′). In the ^13^C-NMR spectrum, one methyl carbon signal *δ*_C_ 21.4 (C-8), one carbonyl carbon signal *δ*_C_ 213.9 (C-2), and a group of sugar carbon signals at *δ*_C_ 102.4, 78.2, 78.0, 74.9, 71.6, and 62.7. Compound **1** was hydrolyzed and derivatized after acid treatment, and GC analysis indicated a retention time consistent with the standard d-glucopyranosyl (d-glucose *t*_R_ = 29.20 min, l-glucose *t*_R_ = 29.56 min), confirming the sugar identification as d-glucopyranosyl moiety. The β-configuration was revealed by the coupling constant *δ*_H_ 4.43 (1H, d, *J* = 7.7 Hz) of the anomeric protons. Preliminary analysis of the data suggested that **1** was a derivative of a monoterpenoid that shared structural skeleton similarities with mudanpioside F [22]. Moreover, the HMBC correlation (Figure 2) from H-1′ to C-4 demonstrated that the glucose was located at the C-4. The pinane-type monoterpenoid skeleton was further established by the HMBC correlation from H-9 to C-1 and C-6, from H_3_-8 to C-5 and C-6, from H-1 to C-2, from H-5 to C-3, and from H-7 to C-1, along with the ^1^H-^1^H COSY correlations (Figure 2) of H-3/H-4/H-5/H-7/H-1. The relative configuration of the **1** was analyzed by the NOESY spectrum (Figure 3). Correlations between H-9b/H-7b, H-7b/H-4, H-1/H_3_-8, and H_3_-8/H-5 indicated that H-1, H-5, and H_3_-8 had a co-facial relationship and were designated as β-oriented. Conversely, H-4 and H-9 were designated as α-oriented. Finally, the absolute configuration of C-1/C-4/C-5/C-6 was assigned as 1*R*/4*S*/5*S*/6*R* by comparing the calculated ECD data (Figure 4) with the experimental data. The combined analysis confirmed that the structure of **1** was identified as Patulumside E. The ^1^H-NMR data of compound **1** were assigned (Table 1). The ^13^C-NMR data of compound **1** were assigned (Table 2).

### 2.3. Characterization of Compound ***2***

Compound **2** was isolated as a colorless solid, with a molecular formula of C_15_H_24_O_8,_ and was examined by HR-ESI-MS (*m*/*z*: 331.1398 [M−H]^−^, calcd. 331.1387; 355.1261 [M+Na]^+^, calcd. 355.1263). Analysis of 1D NMR revealed a similarity with **1**, except that the glucose group was substituted at C-5 and the methyl group was substituted by a hydroxymethyl group at C-6. The sugar unit of **2** was confirmed as β-d-glucopyranosyl using the same method as for **1**. The ^1^H-^1^H COSY spectrum (Figure 2) showed correlations of H-3/H-4 and H-1/H-7. Furthermore, the HMBC spectrum (Figure 2) showed correlations between H_3_-9 and C-5, H_3_-8, and C-1, indicating the presence of the methyl group at C-6. Additionally, the HMBC correlation from H-1′ to C-5 confirmed the position of the sugar group. The relative configuration of **2** was determined by the NOESY correlations (Figure 3) of H-1/H-4 and H-1/H_3_-9. Finally, the absolute configuration of C-1/C-4/C-5 was assigned as 1*R*/4*R*/5*R* by comparing the calculated ECD data (Figure 4) with the experimental data. Therefore, the structure of **2** had been conclusively determined to be Patulumside F. The ^1^H-NMR data of compound **2** were assigned (Table 1). The ^13^C-NMR data of compound **2** were assigned (Table 2).

### 2.4. Characterization of Compound ***3***

Compound **3** was identified as a colorless syrup with the molecular formula of C_16_H_30_O_8_ and was analyzed by HR-ESI-MS (*m*/*z*: 349.1869 [M−H]^−^, calcd. 349.1857; 373.1819 [M+Na]^+^, calcd. 373.1832). The ^1^H-NMR spectrum showed a vinyl moiety signal at *δ*_H_ 5.92 (1H, dd, *J* = 17.4, 10.9 Hz, H-2), 5.33 (1H, dd, *J* = 17.4, 1.6 Hz, H-1a), and 5.17 (1H, dd, *J* = 10.9, 1.6 Hz, H-1b); three methyl signals at *δ*_H_ 1.45 (3H, s, H_3_-9) and 1.19 (6H, s, H_3_-7, 8); two methylene signals at *δ*_H_ 3.90 (1H, d, *J* = 10.0 Hz, H-10a), 3.48 (1H, d, *J* = 10.0 Hz, H-10b), and 1.57 (2H, m, H-4); and one anomeric proton signal at *δ*_H_ 4.29 (1H, d, *J* = 7.6 Hz, H-1′). The ^13^C-NMR spectra demonstrated the presence of a vinyl moiety carbon signal at *δ*_C_ 142.6 (C-2) and 114.4 (C-1); three methyl carbon signals at *δ*_C_ 29.2 (C-7), 29.1 (C-8), and 19.1 (C-9); and a group of sugar carbon signals at *δ*_C_ 105.0, 77.9, 77.8, 75.1, 71.5, and 62.7. By means of gas chromatography (GC), the absolute configuration of the monosaccharide was ascertained to be d-glucopyranosyl. A coupling constant (*J* = 7.6 Hz) was observed for the anomeric proton glu-H-1′, which suggested that glucose was situated in the β-configuration. The ^1^H-^1^H COSY correlations (Figure 2) of H_3_-7/H-6/H_3_-8 and H-1/H-2. In addition, the HMBC spectrum (Figure 2) revealed correlations from H-1 to C-3, which suggested that the vinyl moiety was present at C-3. The correlations from H_3_-7, H_3_-8 to C-5, C-6 and from H_3_-9 to C-4, C-5, C-7, and C-8, respectively, were used to determine the links of methyl. Finally, the monosaccharide was positioned at C-10 by the HMBC correlations from H-1′ to C-10. The NOESY correlations (Figure 3) between H-1/H_3_-9 and H-2/H_3_-9 in the spectrum indicated H_3_-9 and the vinyl group to be in a co-orientation. The absolute configuration of **3** was determined to be 3*S*,5*S* by comparing the experimental CD curve (Figure 4) with the calculated one using ECD calculations. Upon comprehensive analysis, the structure of **3** has been identified as Patulumside G. The ^1^H-NMR data of compound **3** were assigned (Table 1). The ^13^C-NMR data of compound **3** were assigned (Table 2).

### 2.5. Characterization of Compound ***4***

Compound **4** was isolated as a colorless solid, with a molecular formula of C_16_H_28_O_8,_ and was analyzed by HR-ESI-MS (*m*/*z*: 347.1713 [M−H]^−^, calcd. 347.1700; 371.1665 [M+Na]^+^, calcd. 371.1676). The ^1^H-NMR spectrum showed two olefinic signals at *δ*_H_ 5.91 (1H, dd, *J* = 17.4, 10.9 Hz, H-2), 5.39 (1H, ddd, *J* = 8.5, 5.9, 1.4 Hz, H-6), 5.32 (1H, dd, *J* = 17.4, 1.7 Hz, H-1a), and 5.17 (1H, dd, *J* = 10.9, 1.7 Hz, H-1b); one methyl signal at *δ*_H_ 1.64 (3H, s, H_3_-9), four methylene signals at *δ*_H_ 3.90 (2H, m, H-8), 3.89 (1H, d, *J* = 10.0 Hz, H-10a), 3.46 (1H, d, *J* = 10.0 Hz, H-10b), 2.09 (2H, m, H-5), 1.66 (1H, overlapped, H-4a), and 1.55 (1H, ddd, *J* = 13.6, 11.8, 5.2 Hz, H-4b); and one anomeric proton signal at *δ*_H_ 4.26 (1H, d, *J* = 7.7 Hz, H-1′). The ^13^C-NMR spectrum showed two groups of olefinic carbon signals *δ*_C_ 142.4 (C-2), 136.0 (C-7), 126.8 (C-6), and 114.5 (C-1); a methyl carbon signal *δ*_C_ 13.7 (C-9); and a group of sugar carbon signals at *δ*_C_ 105.0, 78.0, 77.9, 75.2, 71.6, and 62.7. The absolute configuration of monosaccharides was determined as d-glucopyranosyl by gas chromatography (GC). The coupling constant of anomeric proton glu-H-1′ (*J* = 7.7 Hz) showed that the glucose was β-positioned. The ^1^H-^1^H COSY spectrum (Figure 2) showed correlations of H-4/H-5/H-6 and H-1/H-2. Furthermore, the HMBC spectrum correlations (Figure 2) from H-1 to C-3 and from H-2 to C-3 confirmed the presence of a vinyl moiety at C-3. Additional HMBC correlations from H-8 and H_3_-9 to C-7 suggested that the positions of the methyl and hydroxymethyl groups were at C-7. The presence of glucose at C-10 was confirmed by the correlations between H-1′ and C-10. The NOESY spectrum (Figure 3) showed correlations between H-6 and H-8, H-5, and H_3_-9, providing further evidence for the *E*-configuration of the olefinic group. Finally, ECD calculations (Figure 4) confirmed that the measured CD curve of **4** matches the simulated curve, establishing the absolute configuration as 3*R*. Based on the overall analysis, the structure of **4** was identified as Patulumside H. The ^1^H-NMR data of compound **4** were assigned (Table 1). The ^13^C-NMR data of compound **4** were assigned (Table 2).

### 2.6. Characterization of Compound ***5***

Compound **5** was analyzed as a colorless solid, with a molecular formula of C_16_H_28_O_8,_ and was analyzed by HR-ESI-MS (*m*/*z*: 393.1765 [M+HCOO]^−^, calcd. 349.1857; 371.1668 [M+Na]^+^, calcd. 371.1676). The ^1^H-NMR spectrum displayed two olefinic proton signals at *δ*_H_ 5.57 (1H, t, *J* = 6.4 Hz, H-7) and 5.49 (1H, td, *J* = 7.2, 1.4 Hz, H-3); two methyl signals at *δ*_H_ 1.71 (3H, s, H_3_-10) and 1.66 (3H, s, H_3_-9); three methylene signals at *δ*_H_ 4.21 (1H, d, *J* = 11.6 Hz, H-1a), 4.07 (1H, d, *J* = 11.6 Hz, H-1b), 4.14 (2H, d, *J* = 6.4 Hz, H-8), and 2.32 (2H, dd, *J* = 7.2, 6.8 Hz, H-4); one oxygenated methine signal at *δ*_H_ 4.01 (1H, t, *J* = 6.8 Hz, H-5); and one anomeric proton signal at *δ*_H_ 4.26 (1H, d, *J* = 7.9 Hz, H-1′). The ^13^C-NMR spectrum showed that **5** had two groups of olefinic carbon signals at *δ*_C_ 140.5 (C-6), 134.6 (C-2), 126.3 (C-7), and 126.1 (C-3), two methyl carbon signals at *δ*_C_ 14.4 (C-10) and 11.8 (C-9), and a group of sugar carbon signals at *δ*_C_ 102.5, 78.2, 77.9, 75.1, 71.8, and 62.8. The absolute configuration of monosaccharides was determined as d-glucopyranosyl by gas chromatography (GC). Further analysis of the 2D NMR of **5** revealed a planar structure identical to that of (2*E*,6*E*,5*R*)-5,8-dihydroxy-2,6-dimethyl-2,6-octadienyl-β-d-glucopyranoside [23], with the only difference being the configuration at C-5. In addition, analysis of the ^1^H-^1^H COSY spectrum correlations (Figure 2) between H-3/H-4/H-5 and H-7/H-8, along with the corresponding HMBC spectrum correlations (Figure 2) from H_3_-9 to C-5 and C-7, from H_3_-10 to C-1 and C-3, from H-8 to C-6 and C-7, and from H-5 to C-4, C-6, and C-9, provided further evidence that the **5** had a monoterpenoid skeleton. The HMBC correlations between H-1′ and C-1 revealed the glucose connected at C-1. In the NOESY spectrum (Figure 3), correlations H-8/H_3_-9, H-5/H-7, H-4/H_3_-10, and H-1/H-3 suggested *E* configurations for the double bonds at C-2, C-3, and C-6, C-7, respectively. ECD calculations (Figure 4) ultimately validated the absolute configuration as 5*S*. Based on the overall analysis, the structure of **5** was identified as Patulumside J. The ^1^H-NMR data of compound **5** were assigned (Table 1). The ^13^C-NMR data of compound **5** were assigned (Table 2).

### 2.7. NO Inhibitory Activities

**Figure 5:**
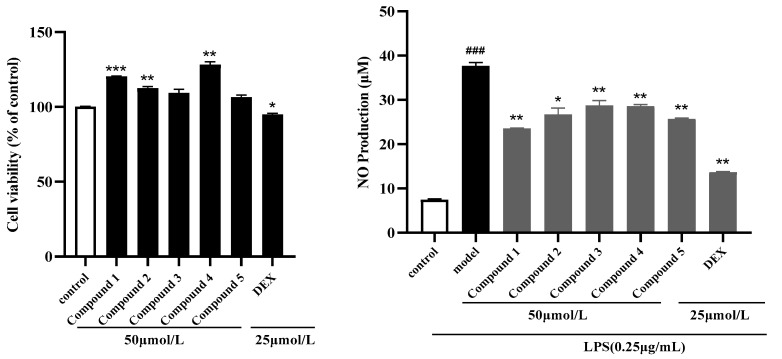
Effects of isolated compounds on cell viability and NO production. * *p* < 0.05, ** *p* < 0.01, *** *p* < 0.001 versus the LPS-treated group; ^###^ *p* < 0.001 versus the control group.

NO was considered as a key inflammatory mediator, which may be helpful to treat the inflammation. To assess the cytotoxicities of the new compounds on RAW 264.7 cells, a CCK-8 assay was conducted. Compared to the Control group, after treatment with 50 μmol/L of the new compound for 24 h, the cell survival rates of compounds **1**–**5** were all above 90%. Therefore, the anti-inflammatory activities of new compounds were investigated at an initial dose of 50 μmol/L by measuring nitric oxide (NO) production in lipopolysaccharide (LPS)-induced RAW264.7 macrophages. The experimental results indicated that compounds **1**–**5** showed moderate inhibitory activity against LPS-stimulated NO production in RAW264.7 cells (Figure 5). 

## 3. Materials and Methods

### 3.1. General Experimental Procedure

HR-ESI-MS spectra were acquired using a Thermo Fisher Q Exactive-Plus spectrometer (Thermo Fisher Scientific, Waltham, MA, USA) with an electrospray ionization source (ESI). Ultra-high-performance liquid chromatography analyses were conducted on a Vanquish horizon spectrometer equipped with a Hypersil gold (2.12 × 250, 1.9 µm) column. NMR spectra were recorded using a BRUKER 600 NEO NMR spectrometer (Brock Co., Ltd., Karlsruhe, Germany). UV spectra were measured on a UV-2700 spectrometer (Shimadzu Co., Ltd., Tokyo, Japan). Optical rotation values were determined using an AUTOPOLⅥ spectrometer (Rudolph Co., Ltd., Lima, OH, USA). IR spectra were recorded on an IR Tracer-100 spectrometer (Shimadzu Co., Ltd., Tokyo, Japan). TLC was performed on silica gel GF254 plates (Qingdao Marine Chemical Ltd., Qingdao, China). Toyopearl HW-40F (Tosoh Corporation, Tokyo, Japan), Sephadex LH-20 (Pharmacia Biotech Co., Ltd., Zurich, Switzerland) were used for column chromatography and silica gel (Qingdao Marine Chemical Co., Ltd., Qingdao, China).

### 3.2. Plant Material 

*Hypericum patulum* Thunb. ex Murray (voucher specimen No. 20180801) was collected from Guiyang, located at 26°18′56″ N and 106°46′9″ E, with an elevation of 1100 m, in Guizhou Province, China during July–August 2018. The plant material was identified as the fresh ripe fruits of *H. patulum*, a member of the Hypericaceae family, by Professor Qingwen Sun at Guizhou University of Traditional Chinese Medicine. The voucher specimen had been deposited at the Guizhou Provincial Key Laboratory of Pharmaceutical Preparations, Guizhou Medical University.

### 3.3. Extraction and Isolation

The fresh ripe fruits of *H. patulum* Thunb. (40 kg) were subjected to extraction using 30% EtOH (*v*/*v*, 390 L) three times. After concentration under reduced pressure, a crude extract (3000 g) was obtained and further separated through a D101 macroporous resin column, with elution performed using water (*v*/*v*, 60 L) and 80% EtOH-H_2_O (*v*/*v*, 180 L). 

The portion eluted with 80% EtOH-H_2_O (1395 g) was subjected to chromatography on a silica gel column (900 g) using a gradient of CHCl_3_-MeOH (each 24 L, 10:0 to 6:4) to yield 11 fractions (Fr. 1–11). Fr. 9 was separated into 9 fractions (Fr. 9.1–9.9) through a silica gel column chromatography eluting with EtOAc-MeOH (9:1 to 5:5). Fr. 9.8 underwent repeated normal-phase silica gel column chromatography (EtOAc-MeOH, 50:1), Toyopearl HW-40F gel column (MeOH), ODS column chromatography (20% MeOH-H_2_O), and silica gel column chromatography (CH_2_Cl_2_-MeOH, 9.5:0.5), resulting in the isolation of 1 (39.8 mg), 2 (6.4 mg), and 3 (150.9 mg). Fr. 10 was purified by Sephadex LH-20 (MeOH), resulted in 11 fractions (Fr. 10.1–10.11). Among them, Fr. 10.3 and Fr. 10.4 were combined and were purified repeatedly by Sephadex LH-20 (MeOH), Toyopearl HW-40F (MeOH), silica gel column chromatography (CH_2_Cl_2_-MeOH 8:2), ODS column chromatography (20% MeOH-H_2_O), and Sephadex LH-20 (50% CH₃COCH₃-H_2_O) to obtain 4 (2.1 mg) and 5 (10.0 mg).

Patulumside E (**1**): colorless solid; [α]: −10.53 (*c* 1.90, MeOH); IR (KBr) *ν*_max_ 3450, 3359, 2927, 1706, 1363, 1176, 1129, 1079, 1030 cm^−1^; ^1^H NMR data, see Table 1; ^13^C NMR data, see Table 2; HR-ESI-MS *m*/*z*: 331.1399 [M−H]^−^, calculated for C_15_H_23_O_8_ 331.1387, 355.1256 [M+Na]^+^, calculated for C_15_H_24_ NaO_8_ 355.1263.

Patulumside F (**2**): colorless solid; [α]: +7.96 (*c* 0.20, MeOH); ^1^H NMR data, see Table 1; ^13^C NMR data, see Table 2; HR-ESI-MS *m*/*z*: 331.1398 [M−H]^−^, calculated for C_15_H_23_O_8_ 331.1387, 355.1261 [M+Na]^+^, calculated for C_15_H_24_ NaO_8_ 355.1263.

Patulumside G (**3**): colorless syrup; [α]: −37.8 (*c* 1.48, MeOH); IR (KBr) *ν*_max_ 3392, 2968, 2930, 2880, 1642, 1380, 1164, 1078, 1039 cm^−1^; ^1^H NMR data, see Table 1; ^13^C NMR data, see Table 2; HR-ESI-MS *m*/*z*: 349.1869 [M−H]^−^, calculated for C_16_H_29_O_8_ 349.1857, 373.1819 [M+Na]^+^, calculated for C_16_H_30_NaO_8_ 373.1832.

Patulumside H (**4**): colorless solid; [α]: −19.05 (*c* 0.21, MeOH); IR (KBr) *ν*_max_ 3412, 2920, 2875, 1632, 1385, 1166, 1078, 1045 cm^−1^; ^1^H NMR data, see Table 1; ^13^C NMR data, see Table 2; HR-ESI-MS *m*/*z*: 347.1713 [M−H]^−^, calculated for C_16_H_27_O_8_ 347.1700, 371.1665 [M+Na]^+^, calculated for C_16_H_28_NaO_8_ 371.1676.

Patulumside J (**5**): colorless solid; [α]: +2.97 (*c* 0.28, MeOH); IR (KBr) *ν*_max_ 3391, 2920, 2876, 1598, 1384, 1160, 1077, 1041, 1020 cm^−1^; ^1^H NMR data, see Table 1; ^13^C NMR data, see Table 2; HR-ESI-MS *m*/*z*: 393.1765 [M+HCOO]^−^, calculated for C_17_H_29_O_10_ 393.1755, 371.1668 [M+Na]^+^, calculated for C_16_H_28_NaO_8_ 371.1676.

### 3.4. Acid Hydrosis and Sugar Identification

Compounds **1**–**5** (0.5 mg) underwent hydrolysis with 2M HCl (2.0 mL) at 95 °C for 3 h. The resulting hydrolysate was cooled and subjected to extraction with ethyl acetate (EtOAc) three times. The aqueous layer was repeatedly evaporated to dryness, reconstituted in pyridine (0.4 mL), and then treated with l-cysteine methyl ester hydrochloride (1.0 mg). The reaction mixture was incubated at 60 °C for 1 h, followed by the addition of trimethylsilyl imidazole (0.15 mL) for another 1 h at 60 °C. The reaction solution was evaporated to dryness under nitrogen, and the residue was dissolved in water (1.0 mL). Extraction with n-hexane (0.5 mL) was performed, and the n-hexane layer was subjected to GC analysis. The absolute configurations of the monosaccharides were confirmed to be D-glucose and L-rhamnose by comparing the retention times with those of authentic samples (*t*_R_ (d-glucose) 29.20 min, *t*_R_ (l-glucose) 29.56 min).

### 3.5. Electronic Circular Dichroism Calculation of Compounds ***1***–***5***

In general, conformational analyses were carried out via random searching in the Sybyl-X 2.0 using the MMFF94S force field with an energy cutoff of 5 kcal/mol [24]. The results showed the six lowest energy conformers. Subsequently, geometry optimizations and frequency analyses were implemented at the B3LYP/6-31G (d)level in CPCM methanol using ORCA5.0.1. All conformers used for property calculations in this work were characterized to be stable points on potential energy surfaces (PES) with no imaginary frequencies. The excitation energies, oscillator strengths, and rotational strengths (velocity) of the first 60 excited states were calculated using the TD-DFT methodology at the PBE0/def2-TZVP level in CPCM methanol using ORCA5.0.1 [25]. The ECD spectra were simulated by the overlapping Gaussian function (half the bandwidth at 1/e peak height, sigma = 0.30 for all) [26]. Gibbs free energies for conformers were determined by using thermal correction at the B3LYP/6-311G(d,p) level and electronic energies evaluated at the wB97M-V/def2-TZVP level in CPCM methanol using ORCA5.0.1. To obtain the final spectra, the simulated spectra of the conformers were averaged according to the Boltzmann distribution theory and their relative Gibbs free energy (∆G). By comparing the experiment spectra with the calculated model molecules, the absolute configuration of the only chiral center was determined. Finally, by comparing the experimental ECD spectra of compounds **1**–**5** with the spectra calculated for the proposed structures using quantum chemical TDDFT, the absolute configurations of the compounds were determined. When the predicted ECD spectra were in good agreement with the experimental spectra, showing similar negative Cotton effects, the absolute configurations were established.

### 3.6. Anti-Inflammatory Assay

The RAW 264.7 macrophage cells were cultured in DMEM containing 10% heat-inactivated fetal calf serum and 1% penicillin/streptomycin at 37 °C in a 5% CO_2_ atmosphere. Cell viability was assessed using the Cell Counting Kit-8 (CCK-8) assay. Compounds **1**–**5** were tested at 50 μmol/L, with those maintaining cell viability above 80% selected for further experiments. RAW 264.7 cells (100 μL) were seeded into a 96-well plate and divided into blank, control, and compound treatment groups. Each group was incubated with the corresponding test solutions for 24 h. After incubation, the cell culture medium was collected, and the optical density (OD) was measured at 450 nm using a Microplate reader. Nitric oxide (NO) levels were determined using a NO Assay Kit according to the manufacturer’s instructions, followed by quantitative analysis [12].

## 4. Conclusions

The phytochemical research on the fresh ripe fruits of *H. patulum* Thunb. resulted in the separation of five monoterpenoid glycosides (1–5), identified with a wide range of spectroscopic methods (^1^H, ^13^C NMR, ^1^H-^1^H COSY, HSQC, HMBC, NOESY, HRESIMS) and physical and chemical methods. Notably, as far as we know, these monoterpene glycosides had not been reported in any other species within the *Hypericum* genus. Our discovery of these monoterpene glycosides significantly enriched the chemical composition of the genus. Preliminary in vitro bioassays indicated that these new compounds exhibited poor NO anti-inflammatory activity. However, the plant glycosides were more polar and often less biologically active than the deconjugated aglycone. The isolation of large quantities of compound 3 facilitates detailed research into its biological activities, including potential functions such as antioxidant, antibacterial, antiviral, and anticancer properties, thereby providing a basis for the development of new drugs or novel bioactive agents. Next, we will explore various aspects of glycoside compounds to discover more bioactive compounds, with the hope of identifying lead compounds.

## Figures and Tables

**Table 1:** ^1^H NMR data (600 MHz; in CD_3_OD of **1**, **2**, **4**, **5**, 400 MHz; in CD_3_OD of **3**).

No	1	2	3	4	5
1a	2.56 t (5.0)	2.41 m	5.33 dd (17.4, 1.6)	5.32 dd (17.4, 1.7)	4.21 d (11.6)
1b			5.17 dd (10.9, 1.6)	5.17 dd (10.9, 1.7)	4.07 d (11.6)
2			5.92 dd (17.4, 10.9)	5.91 dd (17.4, 10.9)	
3a	3.19 overlapped	3.13 dd (19.9, 10.0)			5.49 td (7.2, 1.4)
3b	2.52 dd (20.0, 1.7)	2.39 m			
4a	4.64 m	4.55 dd (10.0, 2.2)	1.57 m	1.66 overlapped	2.32 dd (7.2, 6.8)
4b				1.55 ddd (13.6, 11.8, 5.2)	
5	2.70 m			2.09 (m)	4.01 d (6.8)
6			1.45 overlapped	5.39 ddd (8.5, 5.9, 1.4)	
7a	2.63 m	2.60 dd (10.7, 6.9)	1.19 s		5.57 t (6.4)
7b	1.51 d (10.9)	1.94 d (10.7)			
8	1.44 s	1.13 s	1.19 s	3.90 m	4.14 d (6.4)
9a	3.67 d (11.4)	1.33 s	1.45 s	1.64 s	1.66 s
9b	3.13 d (11.4)				
10a			3.90 d (10.0)	3.89 d (10.0)	1.71 s
10b			3.48 d (10.0)	3.46 d (10.0)	
1′	4.43 d (7.7)	4.42 d (7.8)	4.29 d (7.6)	4.26 d (7.7)	4.26 d (7.9)
2′	3.19 m	3.26 m	3.24 m	3.21 dd (9.2, 7.8)	3.20 dd (9.2, 7.9)
3′	3.35 m	3.29 m	3.30 m	3.27 m	3.35 t (8.9)
4′	3.28 m	3.35 m	3.30 m	3.27 m	3.29 t (9.2)
5′	3.28 m	3.37 m	3.37 m	3.35 m	3.24 ddd (9.2, 5.8, 2.3)
6′a	3.88 dd (12.0, 1.8)	3.82 dd (11.9, 2.4)	3.88 d (12.2)	3.88 dd (11.8, 1.6)	3.87 dd (11.9, 2.3)
6′b	3.66 m	3.69 dd (11.9, 5.0)	3.68 dd (12.2, 6.8)	3.66 dd (11.8, 4.4)	3.67 dd (11.9, 5.7)

**Table 2:** ^13^C NMR data (150 MHz; in CD_3_OD of **1**, **2**, **4**, **5**, 100 MHz; in CD_3_OD of **3**).

No	1	2	3	4	5
1	57.5	53.7	114.4	114.5	75.7
2	213.9	213.9	142.6	142.4	134.6
3	43.0	44.5	76.2	76.1	126.1
4	75.1	69.9	39.0	38.1	34.4
5	43.5	84.0	71.6	22.6	77.7
6	46.0	47.0	45.2	126.8	140.5
7	25.0	30.8	29.2	136.0	126.5
8	21.4	22.8	29.1	69.0	59.2
9	66.9	23.4	19.1	13.7	11.8
10			77.3	77.4	14.4
1′	102.4	99.6	105.0	105.0	102.5
2′	74.9	74.9	75.1	75.2	75.1
3′	78.0	77.7	77.9	78.0	78.2
4′	71.6	71.1	71.5	71.6	71.8
5′	78.2	78.1	77.8	77.9	77.9
6′	62.7	62.2	62.7	62.7	62.8

## Data Availability

All the data in this research were presented in the manuscript and Supplementary Materials.

## Supplementary Materials

The following supporting information can be downloaded at: https://www.mdpi.com/article/10.3390/molecules29133075/s1, Figures S1–S9: 1D/2D NMR, HR-ESIMS, IR, and UV spectra of compounds **1**; Figures S11–S18: 1D/2D NMR, HR-ESIMS, IR, and UV spectra of compounds **2**; Figures S20–S28: 1D/2D NMR, HR-ESIMS, IR, and UV spectra of compounds **3**; Figures S30–S38: 1D/2D NMR, HR-ESIMS, IR, and UV spectra of compounds **4**; Figures S40–S48: 1D/2D NMR, HR-ESIMS, IR, and UV spectra of compounds **4**; Figures S10, S19, S29, S39, S49: GC analysis of compounds **1**–**5**.
