# Supplementary material for: New Monoterpenoid Glycosides from the Fruits of Hypericum patulum Thunb
Source: Molecules. 2024 Jun 27;29(13):3075. doi: 10.3390/molecules29133075 (PMC11243496; doi:10.3390/molecules29133075)
Supplement: Supplementary file 1 [file molecules-29-03075-s001.zip › supplementary-3004295.pdf]

New Monoterpenoid Glycosides From the fruits of *Hypericum patulum* Thunb.

Li Jiang [1,2,†], Xue Ma [1,2,3,†], Yang Wang [2,4], Jian-Ping Yang [2], Yong Huang [2,4,5], Chun-Hua Liu [1,2,*] and Yong-Jun Li [1,2,4,5,*]

[1] Engineering Research Center for the Development and Application of Ethnic Medicine and TCM, Ministry of Education/Guizhou Provincial Engineering Research Center for the Development and Application of Ethnic Medicine and TCM, Guizhou Medical University, Guiyang 550004, China; jiangli0324@gmc.edu.cn (L.J.); xuema0111@163.com (X.M.)
[2] School of Pharmacy, Guizhou Medical University, Guiyang 550004, China; 15180803607@163.com (Y.W.); yangjianping762@gmail.com (J.-P.Y.); huangy2020@126.com (Y.H.)
[3] School of Basic Medical Sciences, Guizhou Medical University, Guiyang 550004, China
[4] Guizhou Provincial Key Laboratory of Pharmaceutics/State Key Laboratory of Functions and Applications of Medicinal Plants, Guizhou Medical University, Guiyang 550004, China
[5] National Engineering Research Center of Miao's Medicines, Guizhou Medical University, Guiyang 550004, China
* Correspondence: liuch2016@gmc.edu.cn (C.-H.L.); liyongjun026@gmc.edu.cn (Y.-J.L.)
† These authors contributed equally to this work.

Contents

Figure S1. HR-ESI-MS spectrum of compound **1**.

Figure S2. UV spectrum of compound **1**.

Figure S3. IR spectrum of compound **1**.

Figure S4. ^{1}H-NMR (600MHz, CD_3OD) spectrum of compound **1**.

Figure S5. ^{13}C-NMR (150MHz, CD_3OD) spectrum of compound **1**.

Figure S6. HMQC spectrum of compound **1**.

Figure S7. HMBC spectrum of compound **1**.

Figure S8. ^{1}H-^{1}H COSY spectrum of compound **1**.

Figure S9. NOESY spectrum of compound **1**.

Figure S10. GC spectrum of saccharides hydrolysis and derivatization of compound **1.**

Figure S11. HR-ESI-MS spectrum of compound **2**.

Figure S12. UV spectrum of compound **2**.

Figure S13. ^{1}H-NMR (600MHz, CD_3OD) spectrum of compound **2**.

Figure S14. ^{13}C-NMR (150MHz, CD_3OD) spectrum of compound **2**.

Figure S15. HMQC spectrum of compound **2**.

Figure S16. HMBC spectrum of compound **2**.

Figure S17. ^{1}H-^{1}H COSY spectrum of compound **2**.

Figure S18. NOESY spectrum of compound **2**.

Figure S19. GC spectrum of saccharides hydrolysis and derivatization of compound **2.**

Figure S20. HR-ESI-MS spectrum of compound **3**.

Figure S21. UV spectrum of compound **3**.

Figure S22. IR spectrum of compound **3**.

Figure S23. ^{1}H-NMR (400MHz, CD_3OD) spectrum of compound **3**.

Figure S24. ^{13}C-NMR (100MHz, CD_3OD) spectrum of compound **3**.

Figure S25. HMQC spectrum of compound **3**.

Figure S26. HMBC spectrum of compound **3**.

Figure S27. ^{1}H-^{1}H COSY spectrum of compound **3**.

Figure S28. NOESY spectrum of compound **3**.

Figure S29. GC spectrum of saccharides hydrolysis and derivatization of compound **3.**

Figure S30. HR-ESI-MS spectrum of compound **4**.

Figure S31. UV spectrum of compound **4**.

Figure S32. IR spectrum of compound **4**.

Figure S33. ^{1}H-NMR (600MHz, CD_3OD) spectrum of compound **4**.

Figure S34. ^{13}C-NMR (150MHz, CD_3OD) spectrum of compound **4**.

Figure S35. HMQC spectrum of compound **4**.

Figure S36. HMBC spectrum of compound **4**.

Figure S37. ^{1}H-^{1}H COSY spectrum of compound **4**.

Figure S38. NOESY spectrum of compound **4**.

Figure S39. GC spectrum of saccharides hydrolysis and derivatization of compound **4.**

Figure S40. HR-ESI-MS spectrum of compound **5**.

Figure S41. UV spectrum of compound **5**.

Figure S42. IR spectrum of compound **5**.

Figure S43. ^{1}H-NMR (600MHz, CD_3OD) spectrum of compound **5**.

Figure S44. ^{13}C-NMR (150MHz, CD_3OD) spectrum of compound **5**.

Figure S45. HMQC spectrum of compound **5**.

Figure S46. HMBC spectrum of compound **5**.

Figure S47. ^{1}H-^{1}H COSY spectrum of compound **5**.

Figure S48. NOESY spectrum of compound **5**.

Figure S49. GC spectrum of saccharides hydrolysis and derivatization of compound **5.**

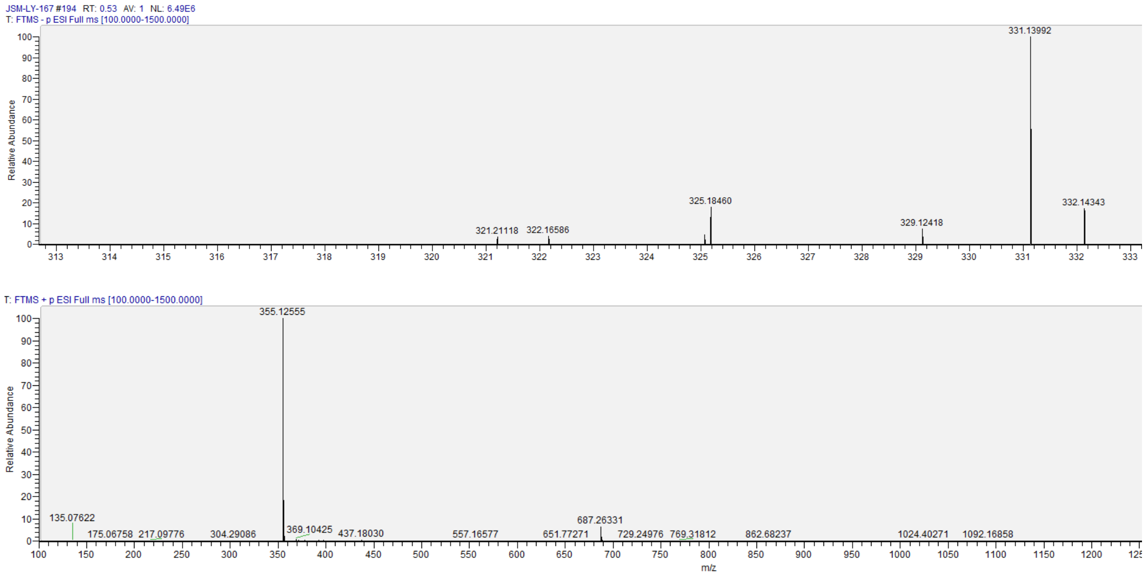

Figure S1. HR-ESI-MS spectrum of compound **1**.

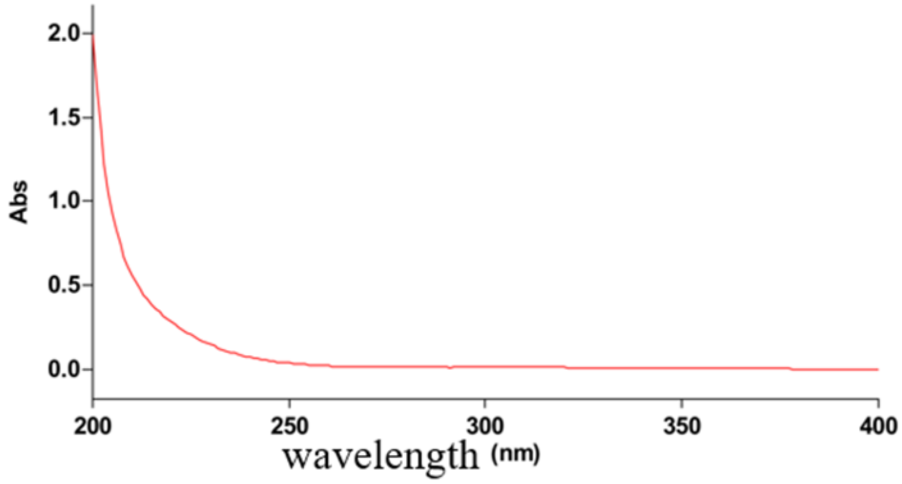

Figure S2. UV spectrum of compound **1**.

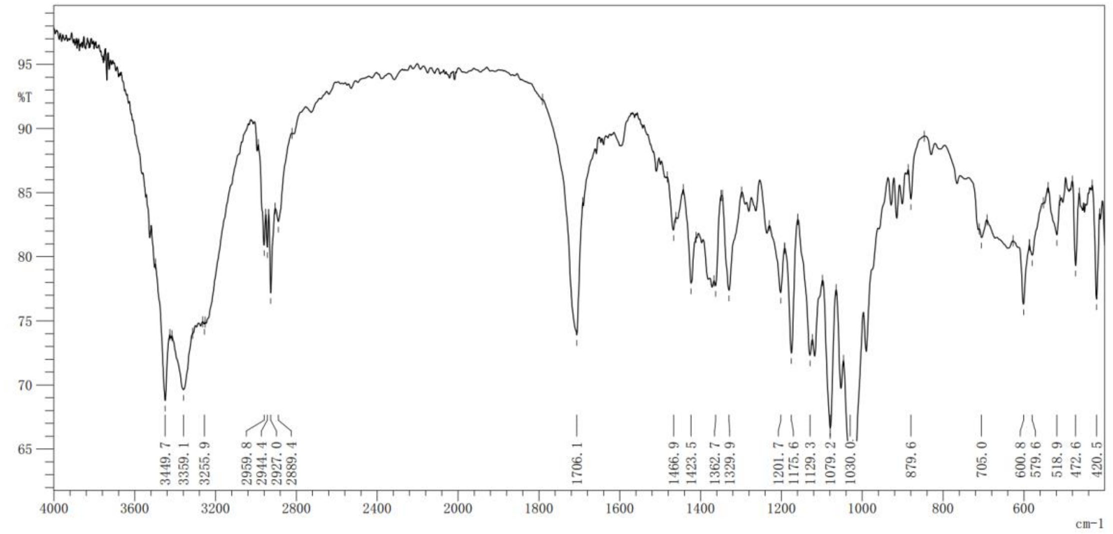

Figure S3. IR spectrum of compound **1**.

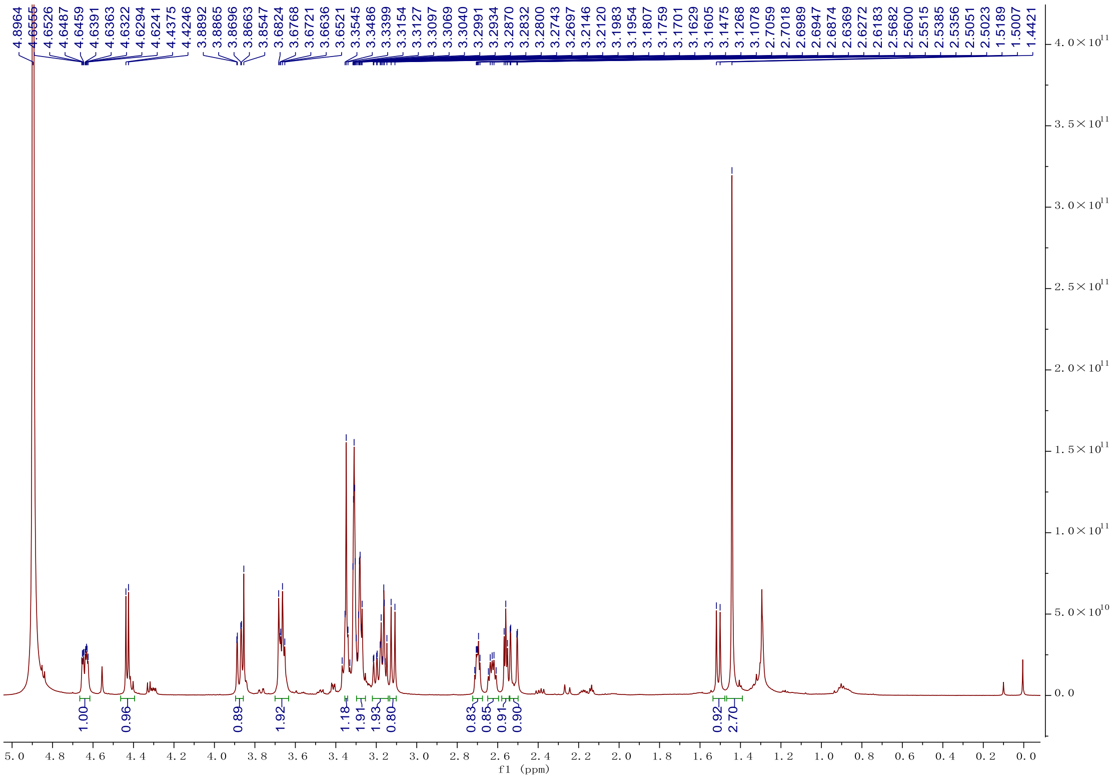

Figure S4. ^{1}H-NMR (600MHz, CD_3OD) spectrum of compound **1**.

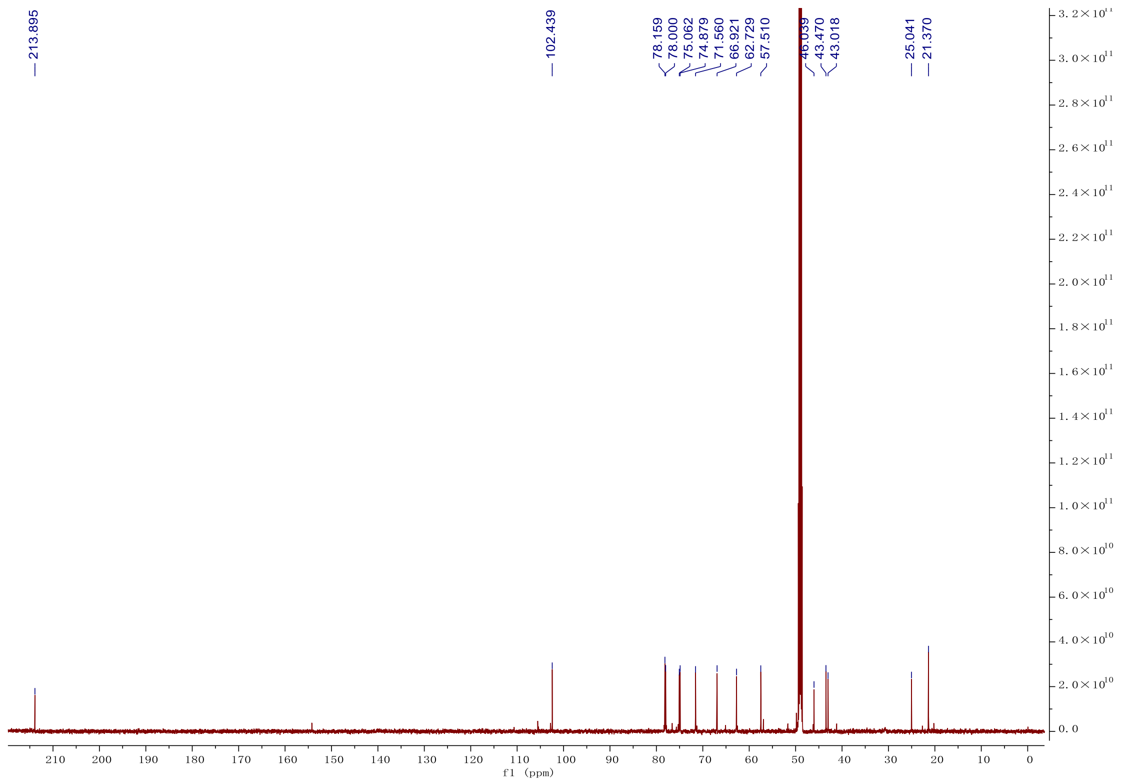

Figure S5. ^{13}C-NMR (150MHz, CD_3OD) spectrum of compound **1**.

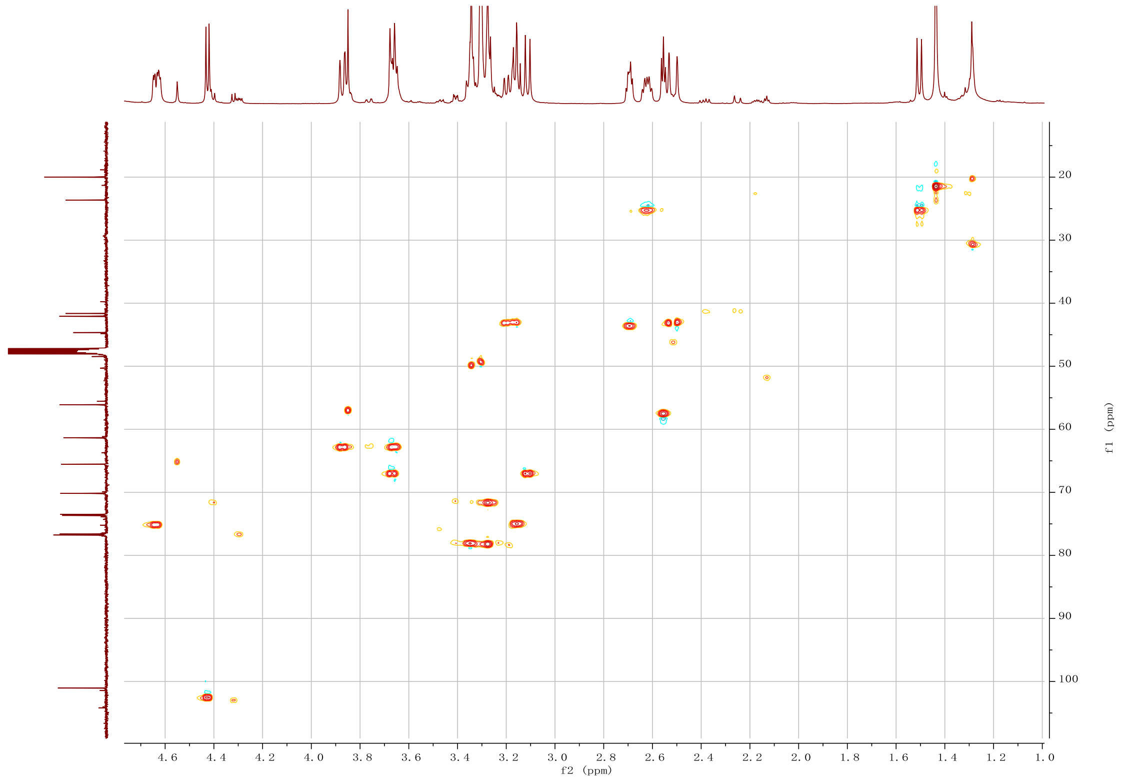

Figure S6. HMQC spectrum of compound **1**.

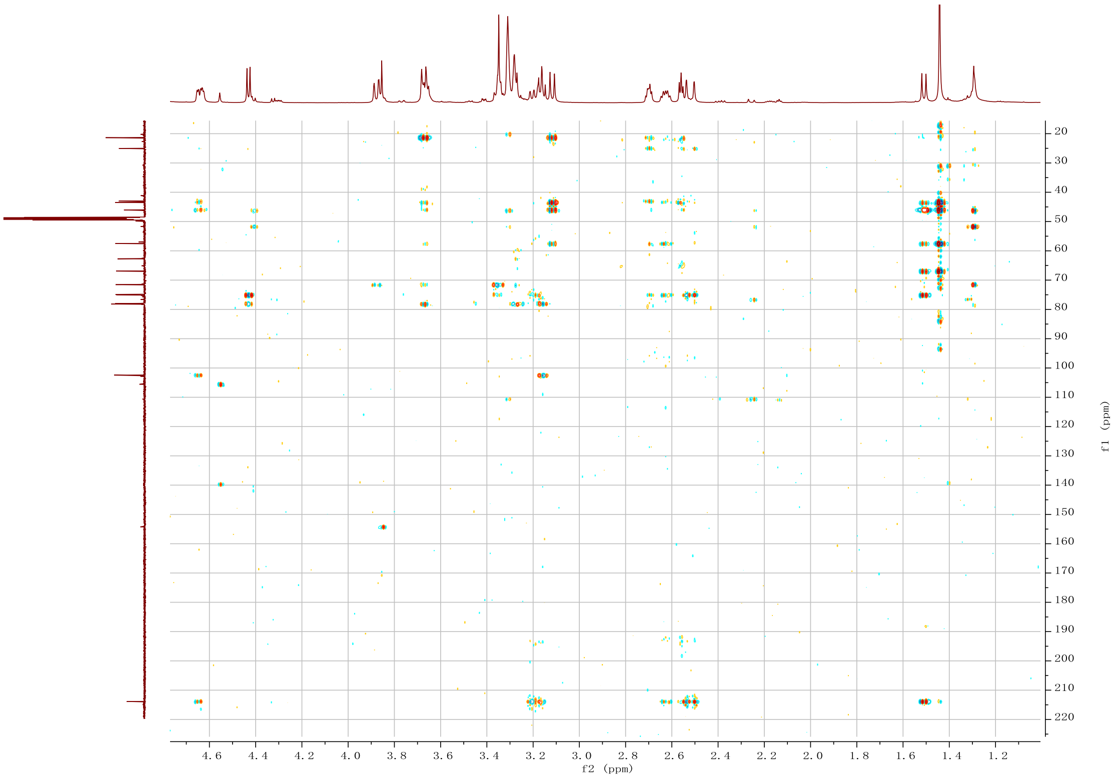

Figure S7. HMBC spectrum of compound **1**.

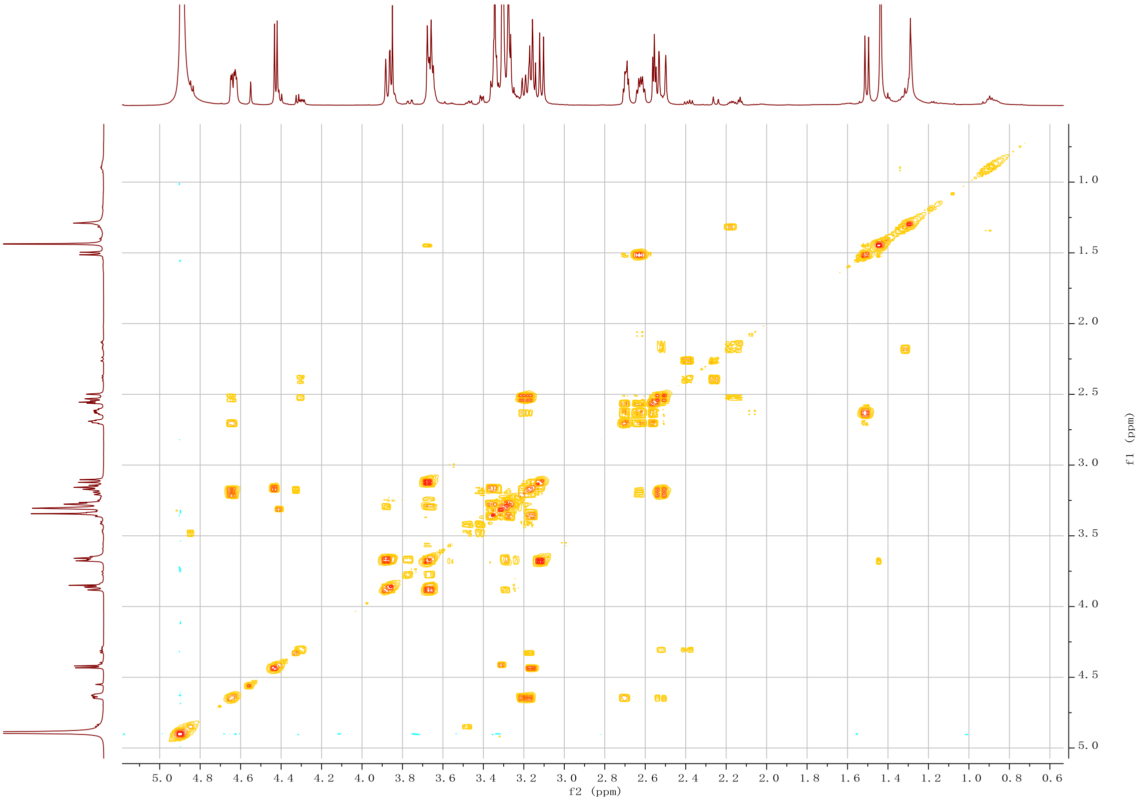

Figure S8. ^{1}H-^{1}H COSY spectrum of compound **1**.

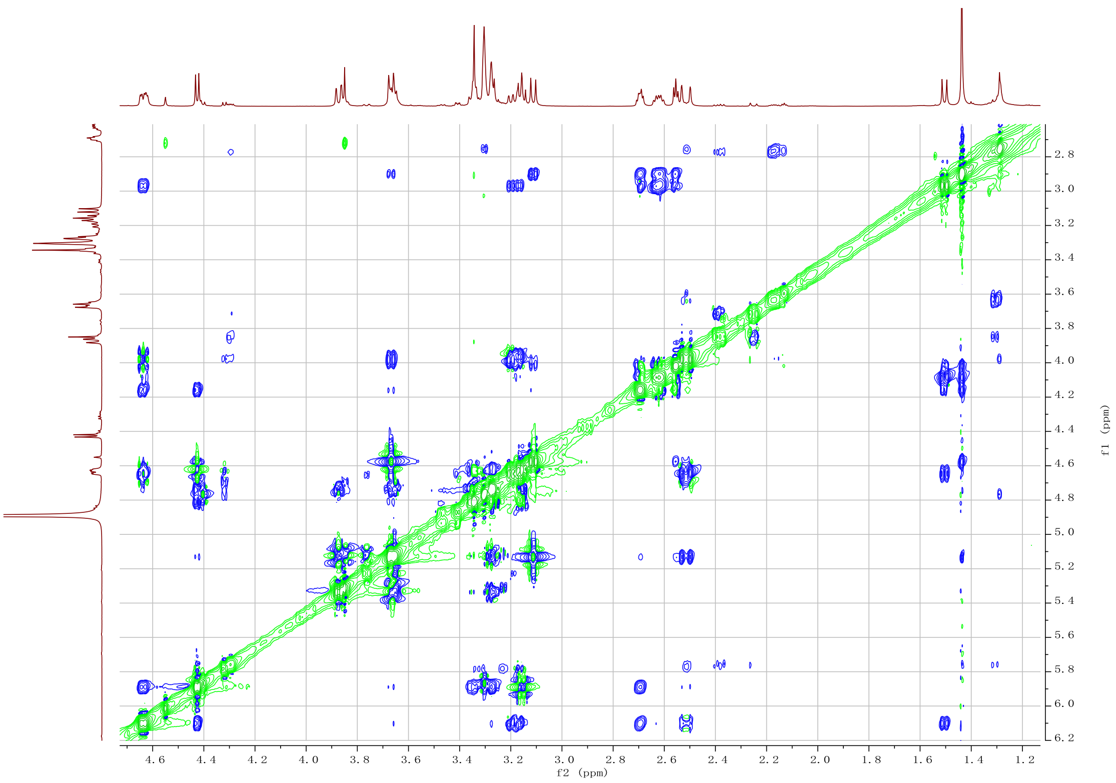

Figure S9. NOESY spectrum of compound **1**.

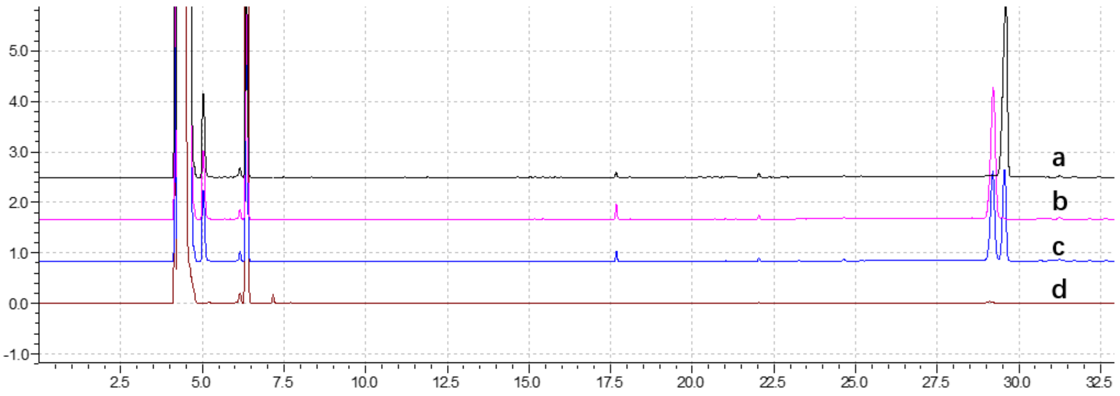

Figure S10. GC spectrum of saccharides hydrolysis and derivatization of compound **1.**

(a. derivatives of L glucose, t_R L-glucose derivative 29.56 min; b. derivatives of standard D, t_R D-glucose derivative 29.20 min; c. derivatives of standard D and L glucose, d. acid hydrolysis derivative of compound **1**.)

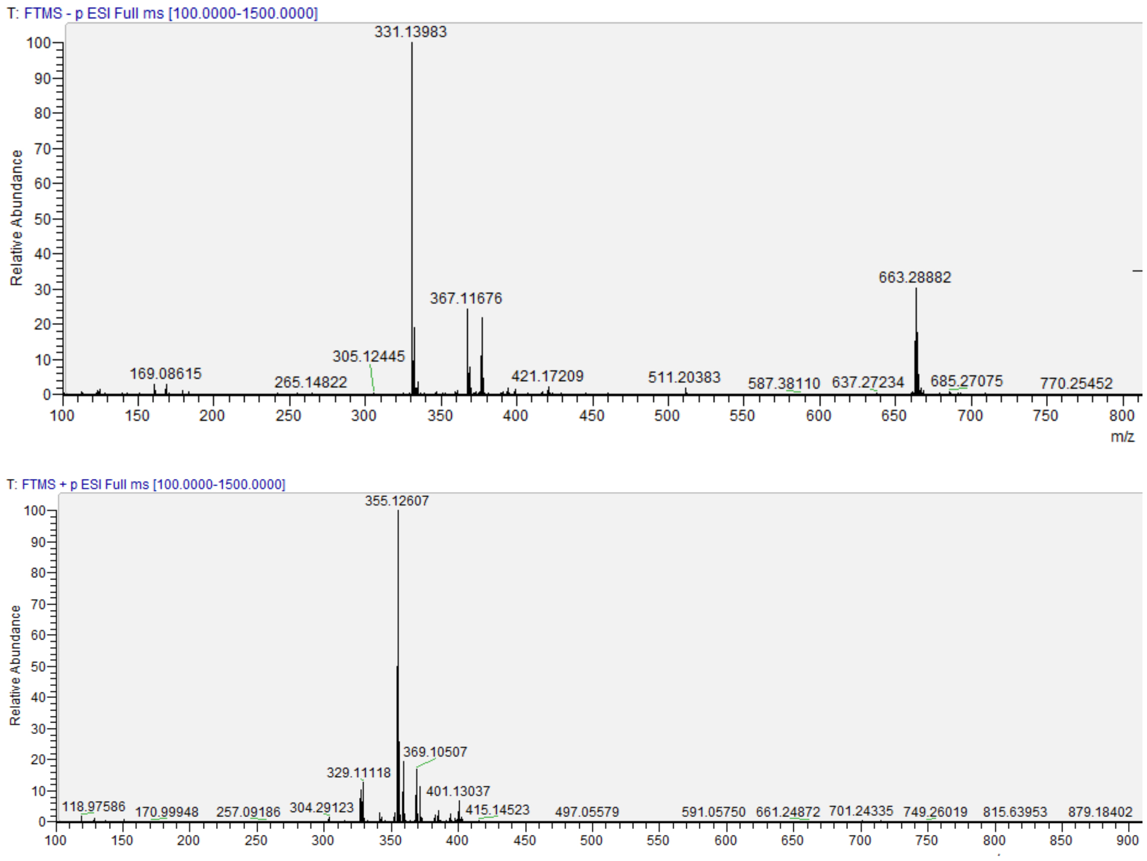

Figure S11. HR-ESI-MS spectrum of compound **2**.

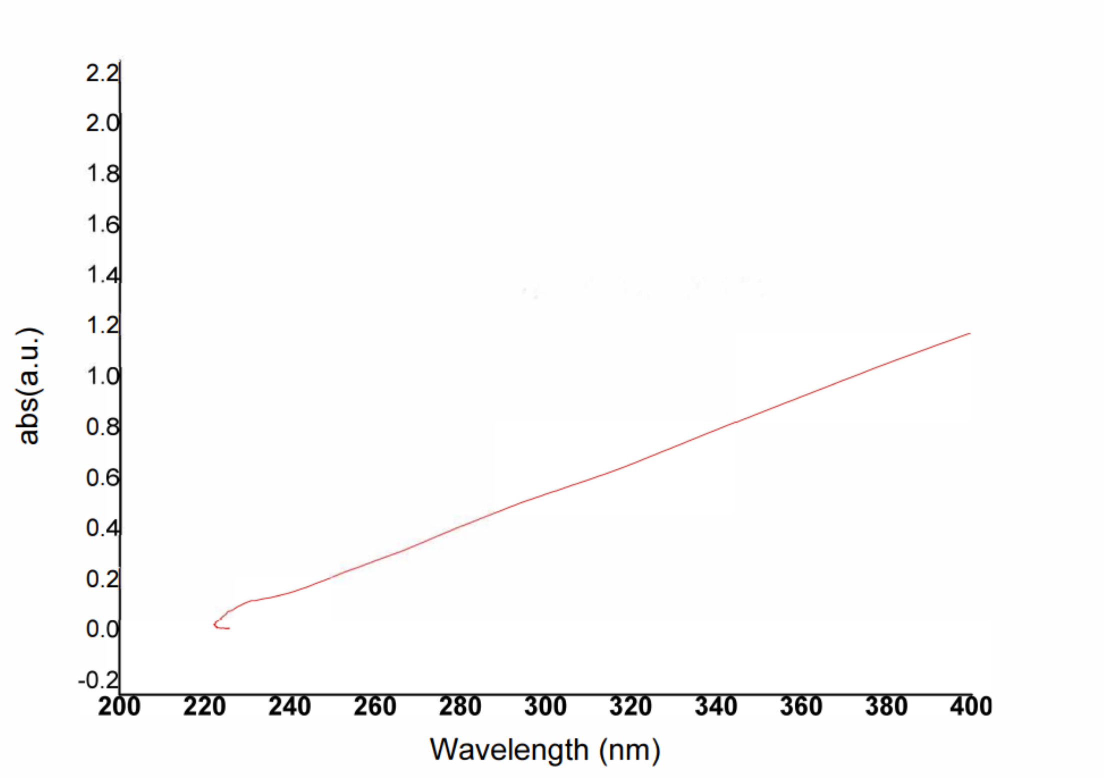

Figure S12. UV spectrum of compound **2**.

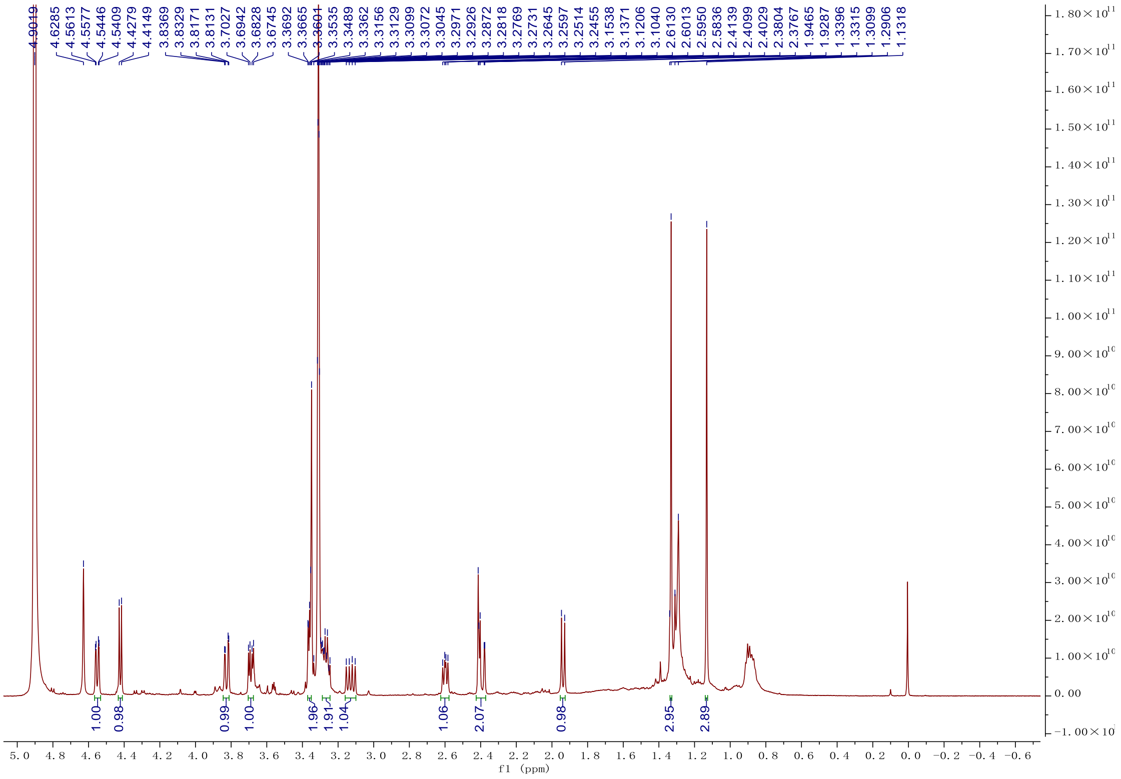

Figure S13. ^{1}H-NMR (600MHz, CD_3OD) spectrum of compound **2**.

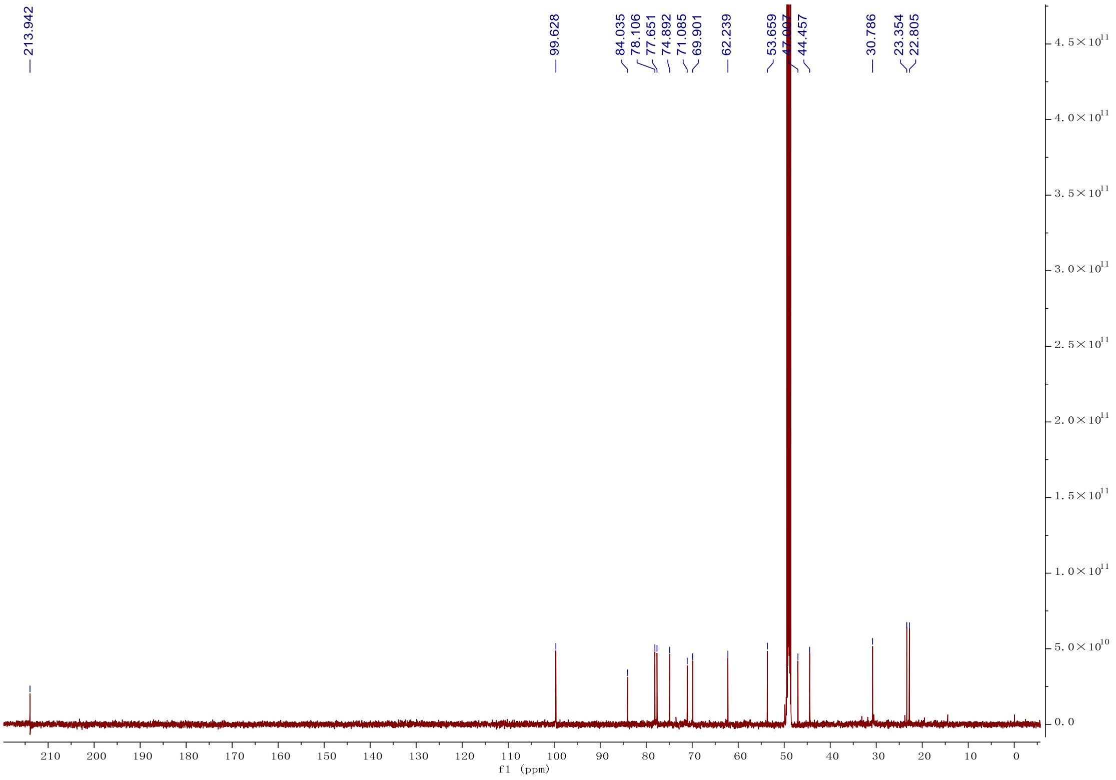

Figure S14. ^{13}C-NMR (150MHz, CD_3OD) spectrum of compound **2**.

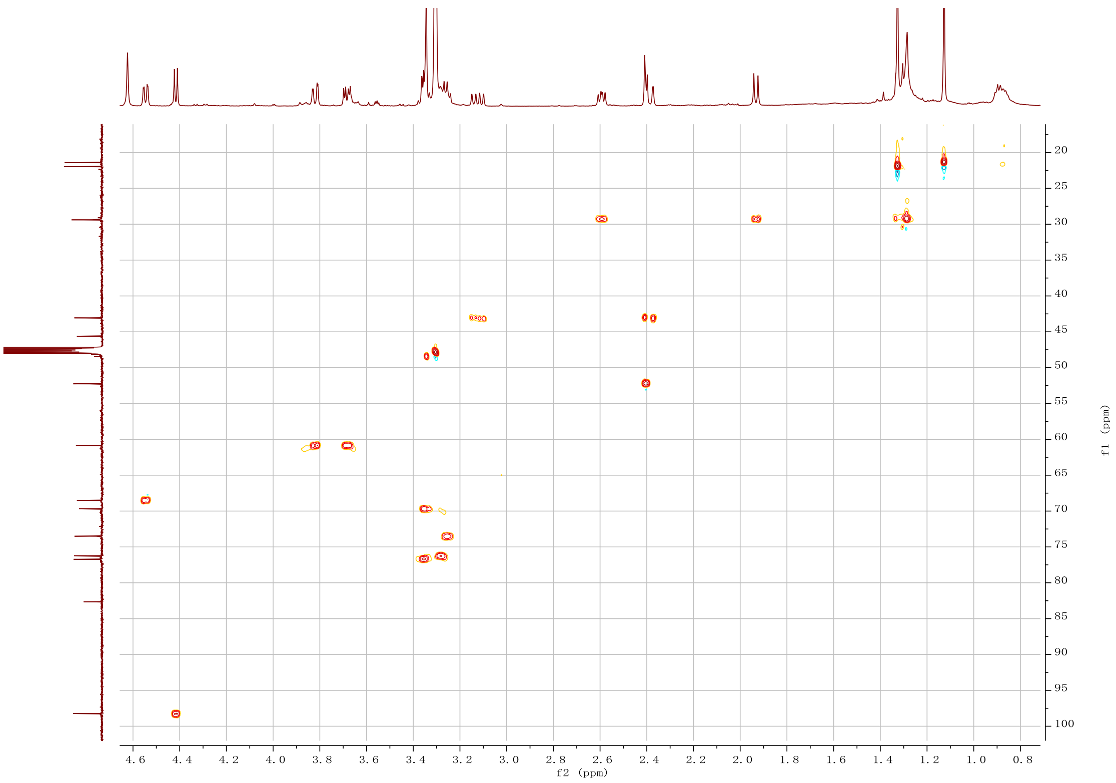

Figure S15. HMQC spectrum of compound **2**.

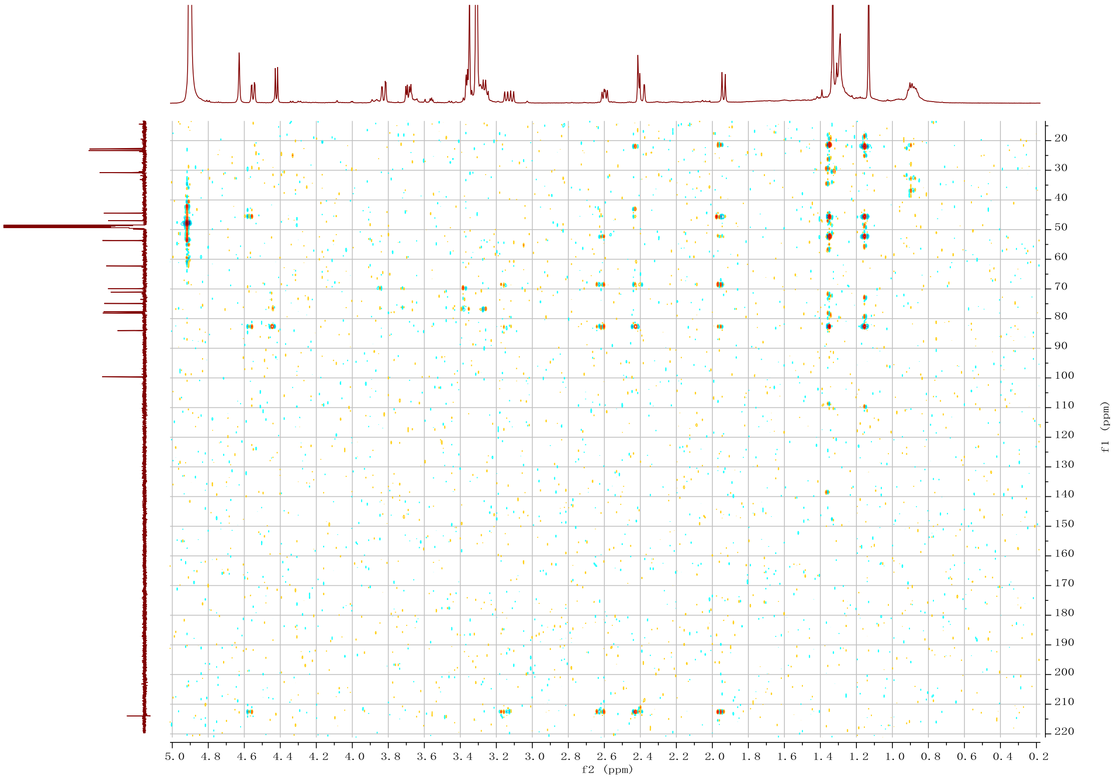

Figure S16. HMBC spectrum of compound **2**.

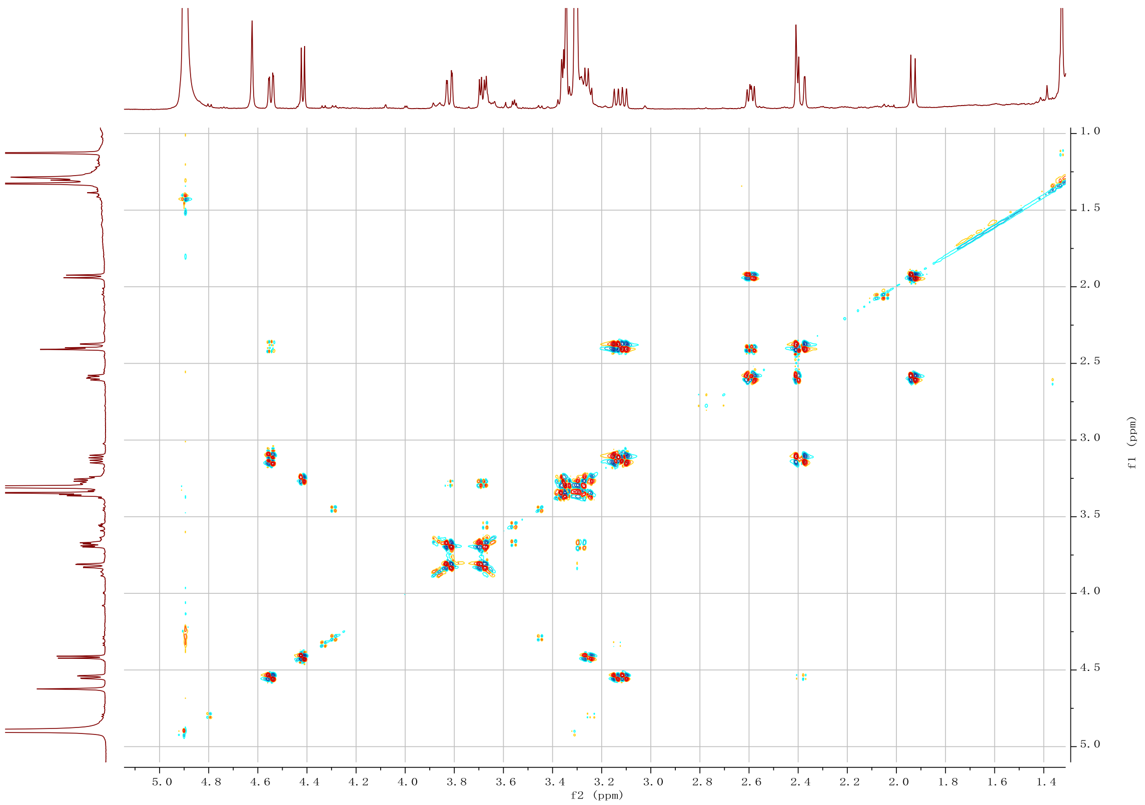

Figure S17. ^{1}H-^{1}H COSY spectrum of compound **2**.

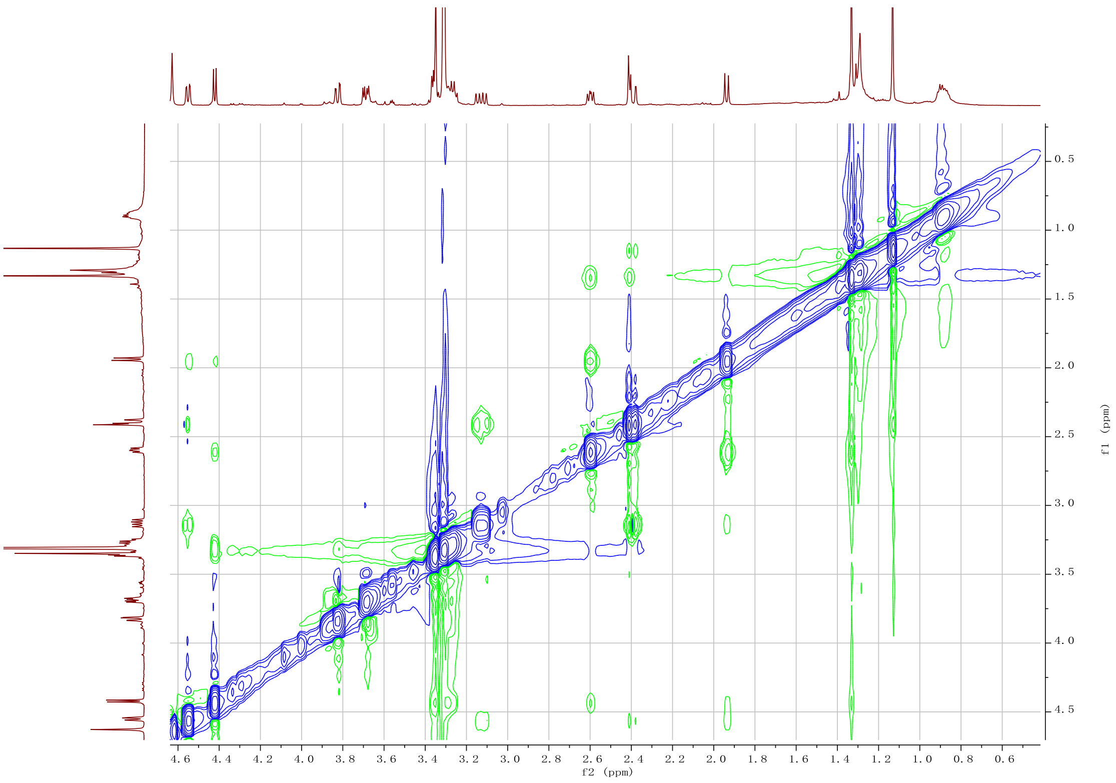

Figure S18. NOESY spectrum of compound **2**.

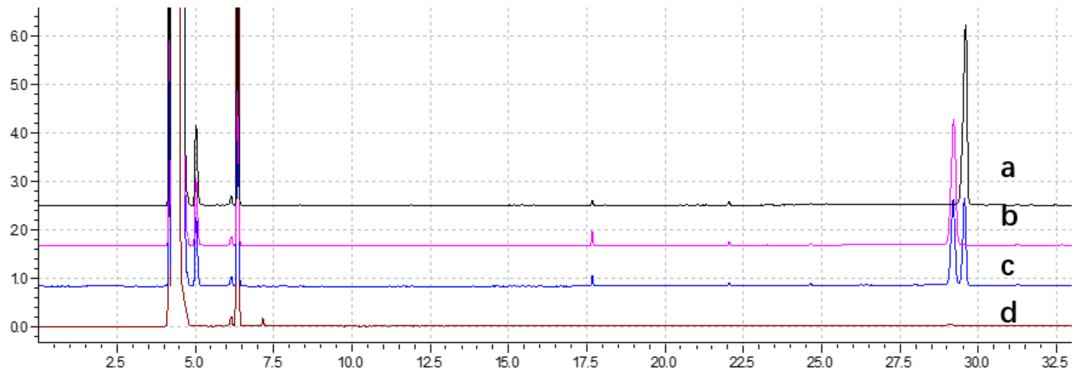

Figure S19. GC spectrum of saccharides hydrolysis and derivatization of compound **2.**

(a. derivatives of L glucose, t_R L-glucose derivative 29.56 min; b. derivatives of standard D, t_R D-glucose derivative 29.20 min; c. derivatives of standard D and L glucose, d. acid hydrolysis derivative of compound **2**.)

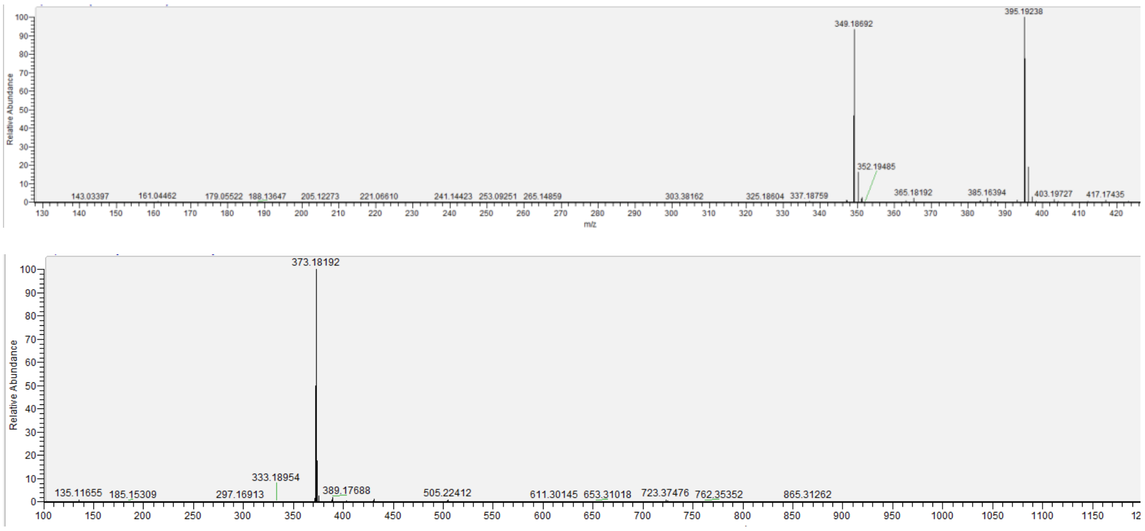

Figure S20. HR-ESI-MS spectrum of compound **3**.

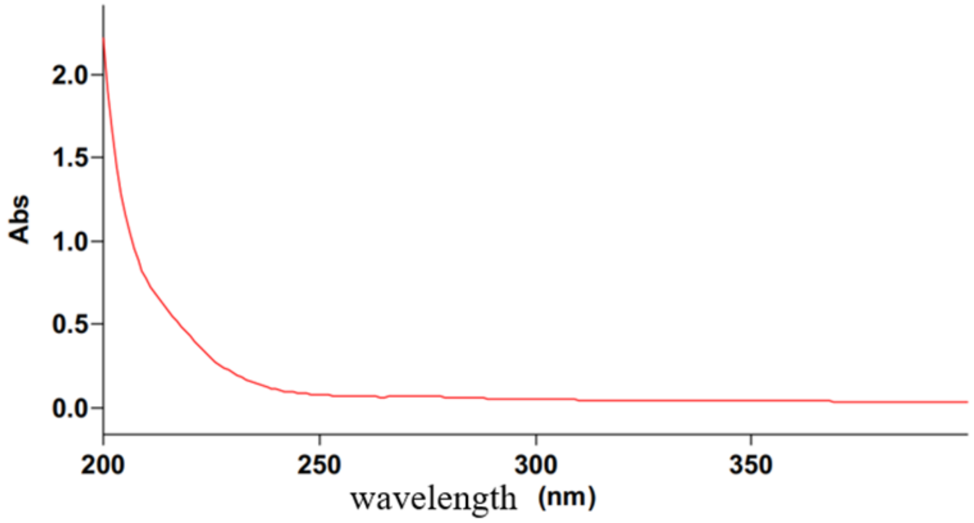

Figure S21. UV spectrum of compound **3**.

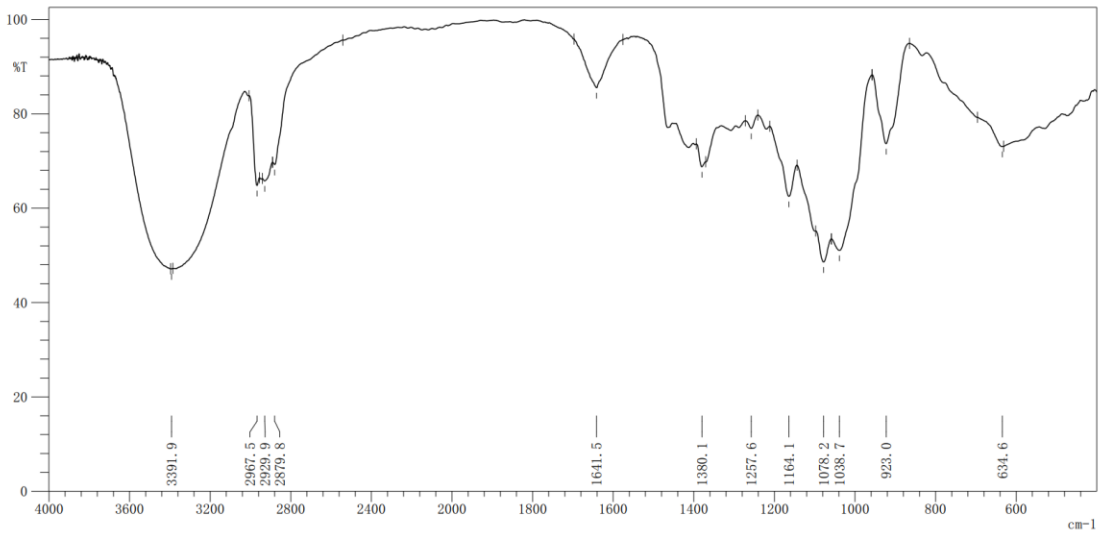

Figure S22. IR spectrum of compound **3**.

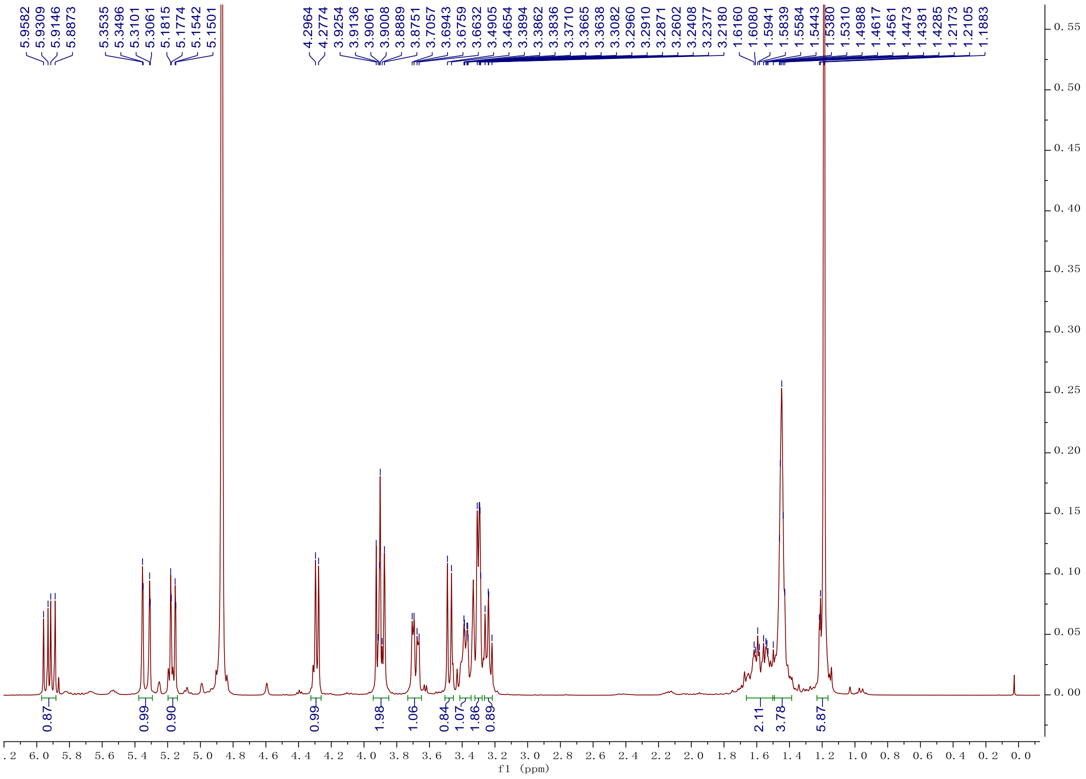

Figure S23. ^{1}H-NMR (400MHz, CD_3OD) spectrum of compound **3**.

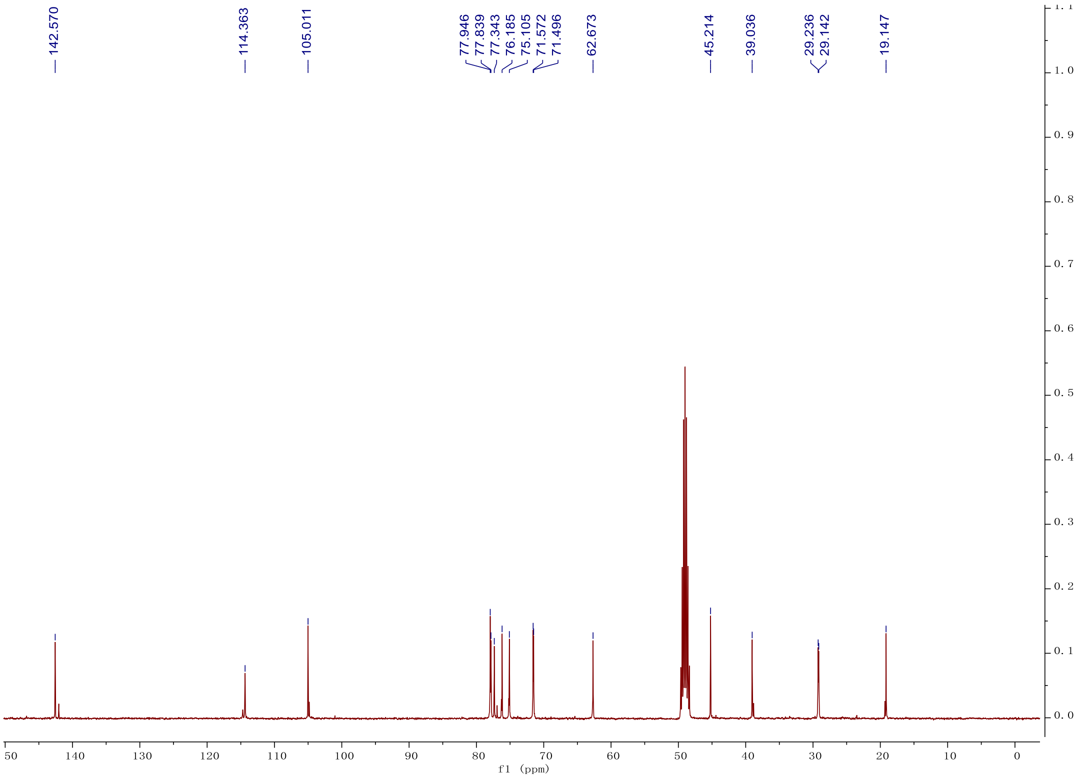

Figure S24. ^{13}C-NMR (100MHz, CD_3OD) spectrum of compound **3**.

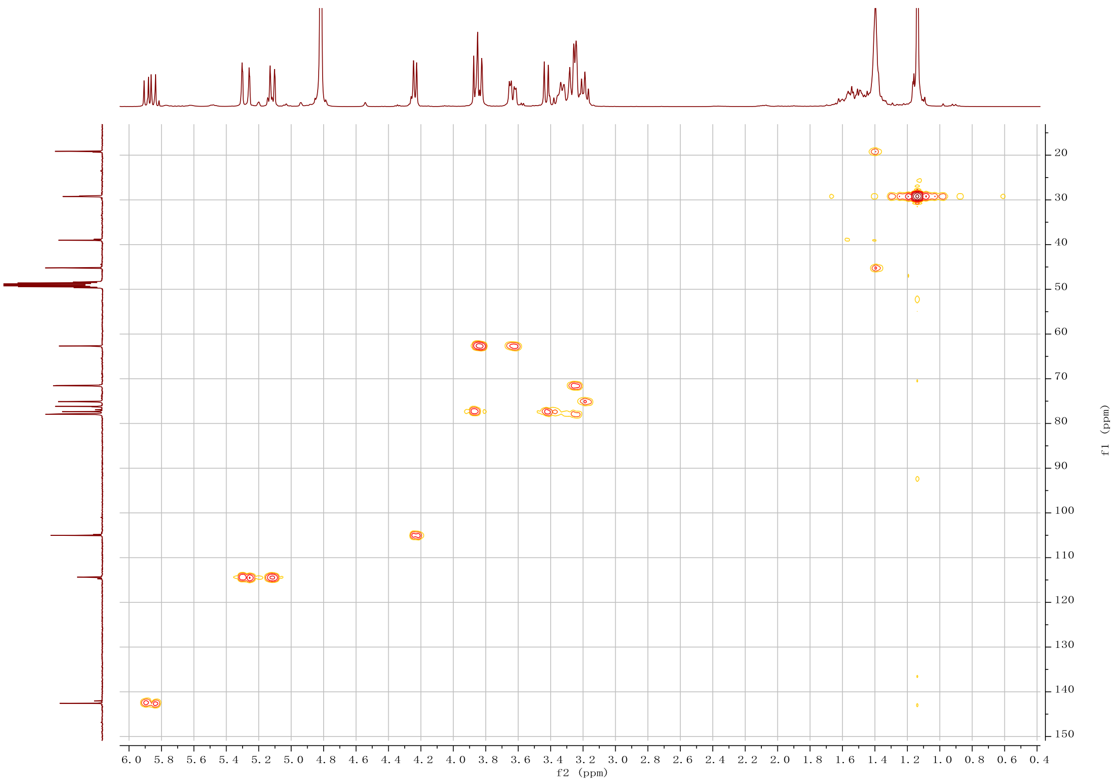

Figure S25. HMQC spectrum of compound **3**.

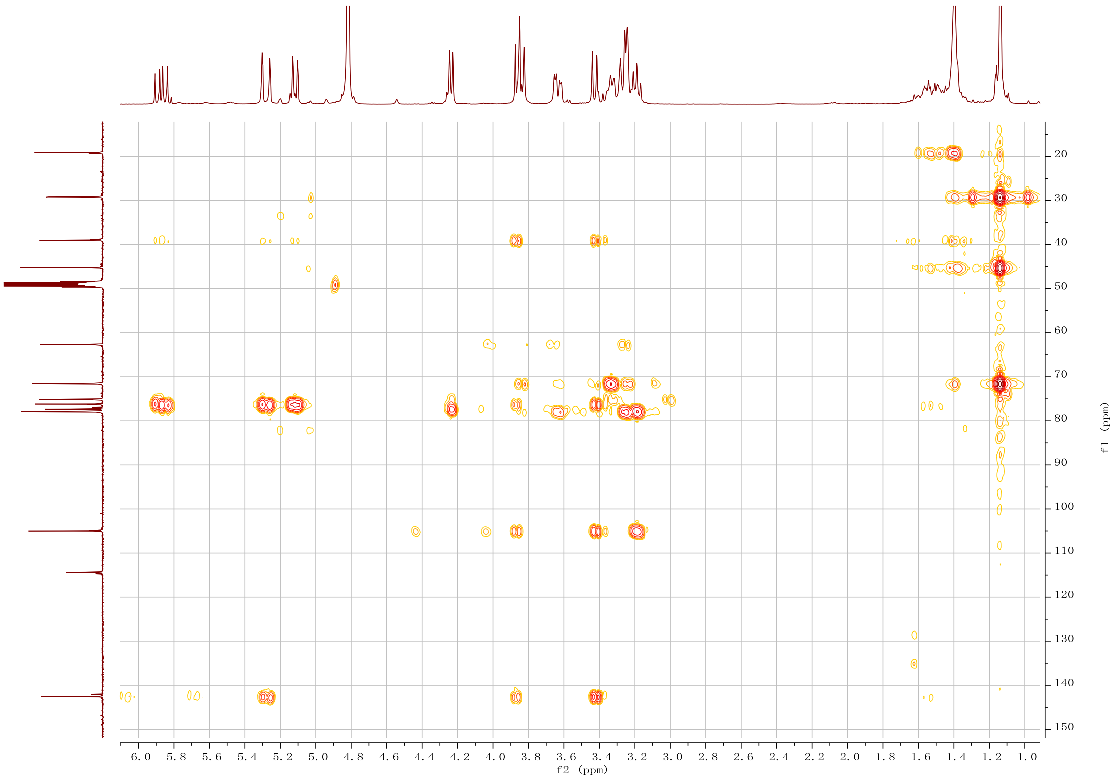

Figure S26. HMBC spectrum of compound **3**.

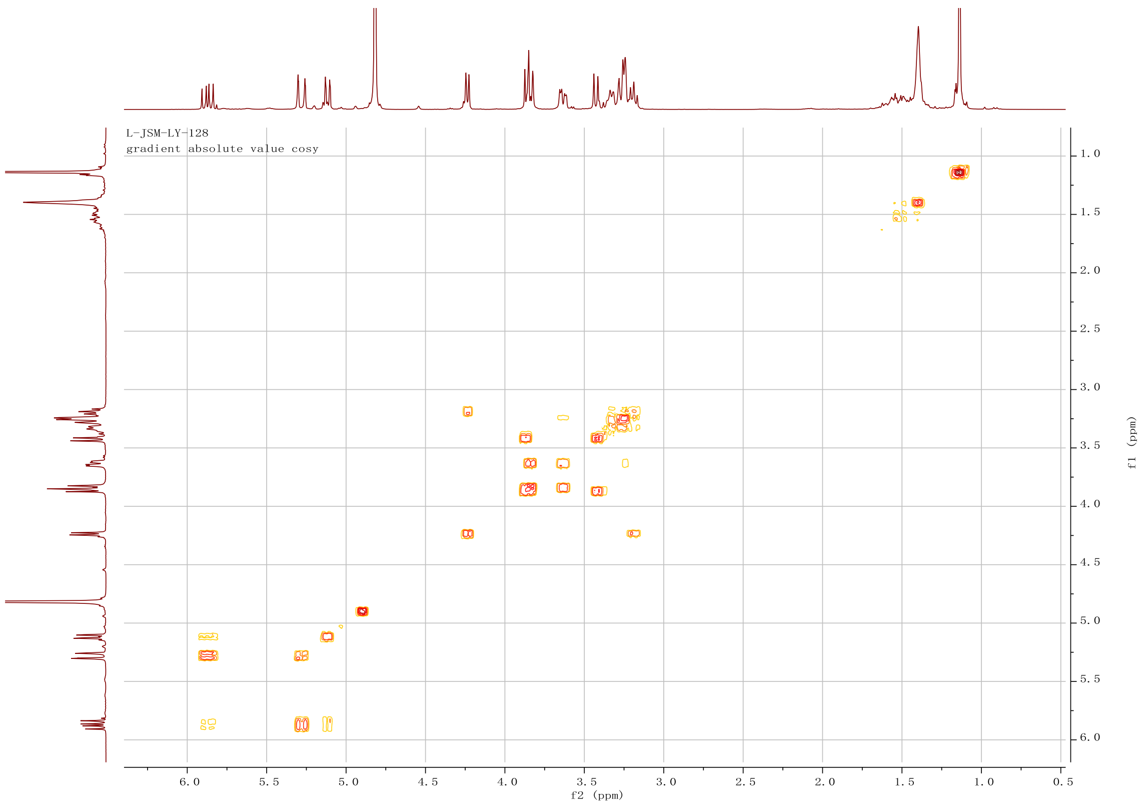

Figure S27. ^{1}H-^{1}H COSY spectrum of compound **3**.

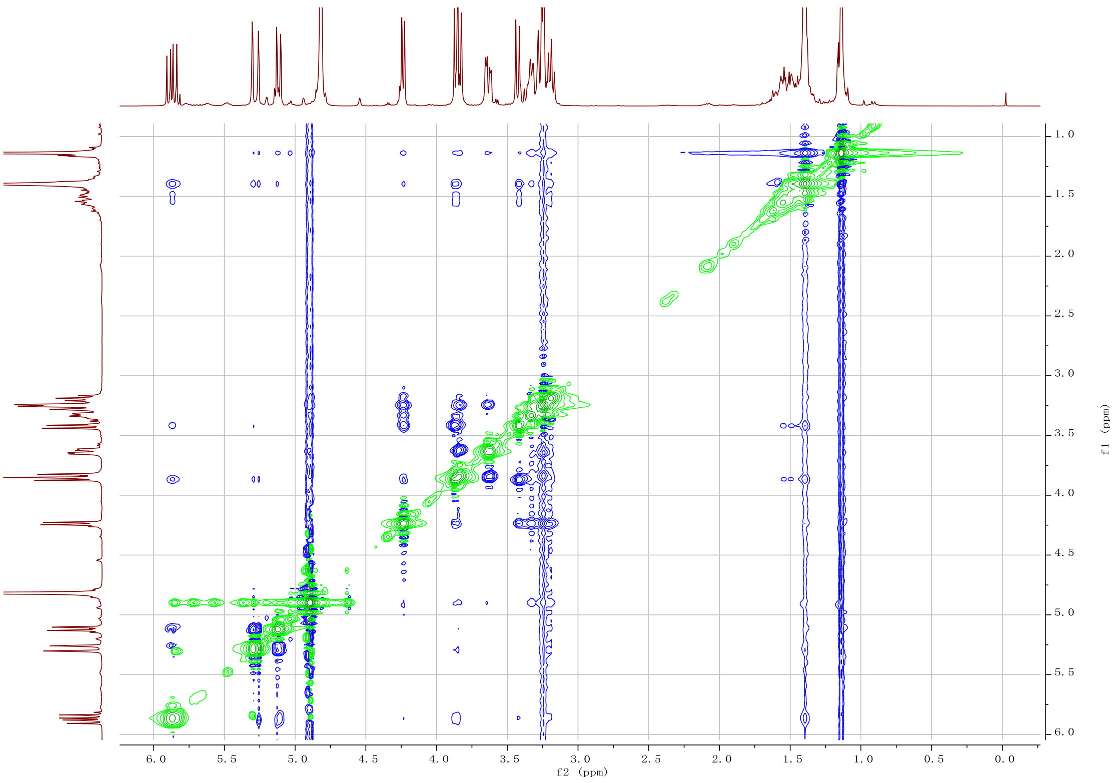

Figure S28. NOESY spectrum of compound **3**.

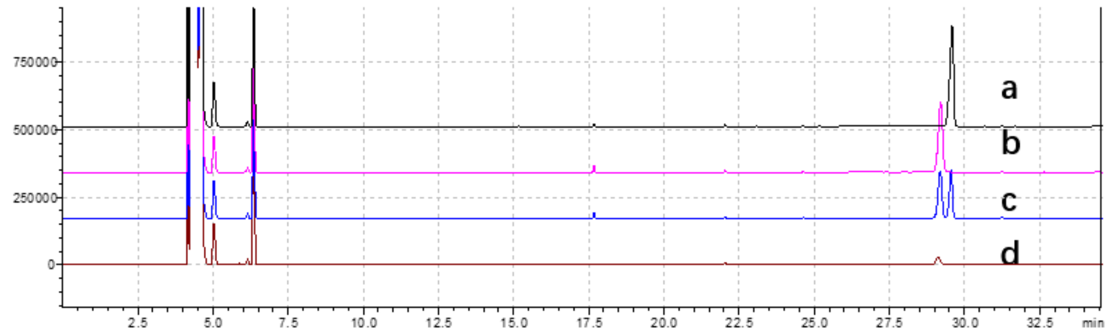

Figure S29. GC spectrum of saccharides hydrolysis and derivatization of compound **3.**

(a. derivatives of L glucose, t_R L-glucose derivative 29.56 min; b. derivatives of standard D, t_R D-glucose derivative 29.20 min; c. derivatives of standard D and L glucose, d. acid hydrolysis derivative of compound **3**.)

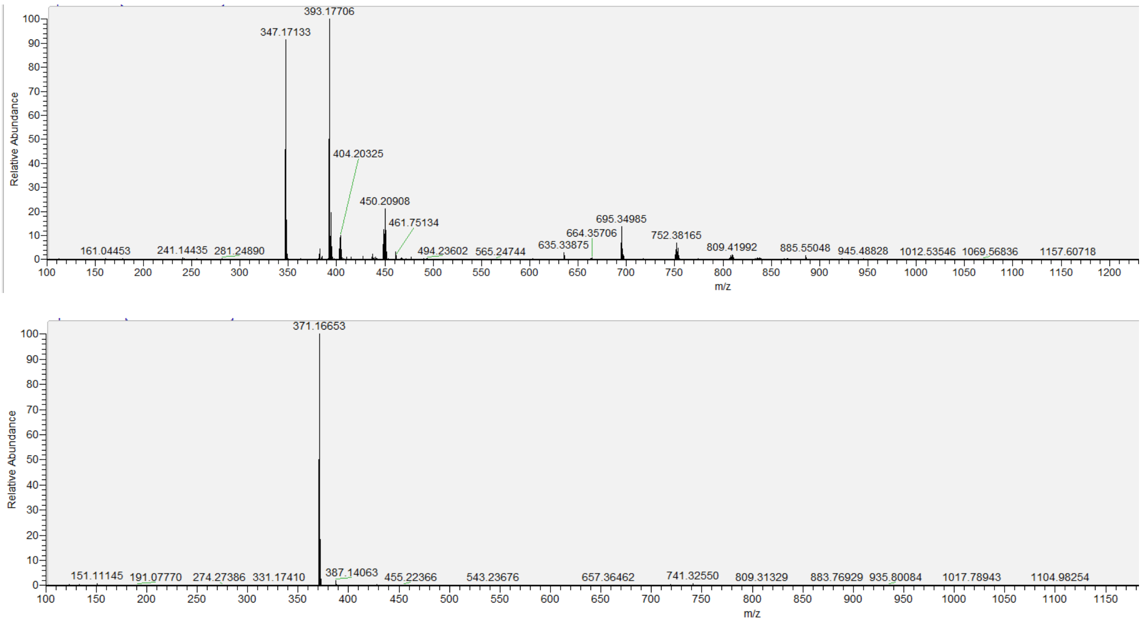

Figure S30. HR-ESI-MS spectrum of compound **4**.

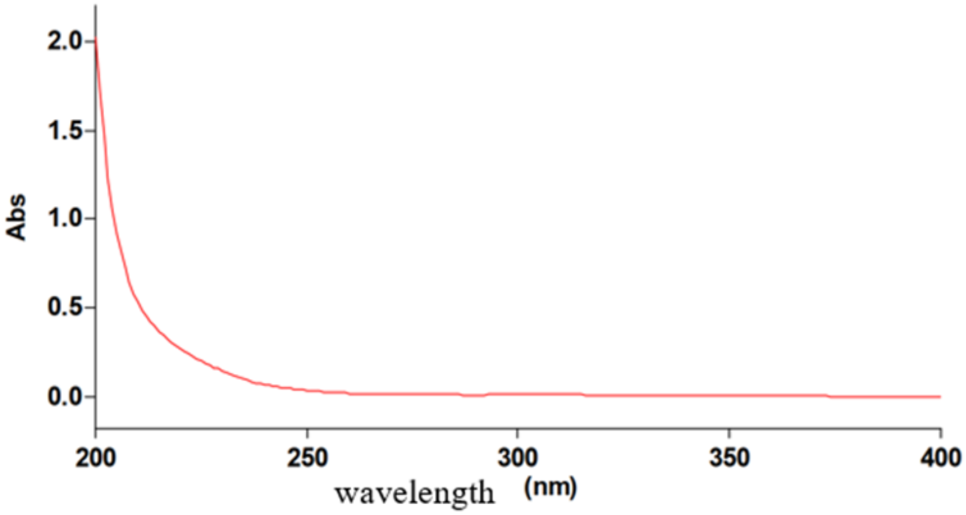

Figure S31. UV spectrum of compound **4**.

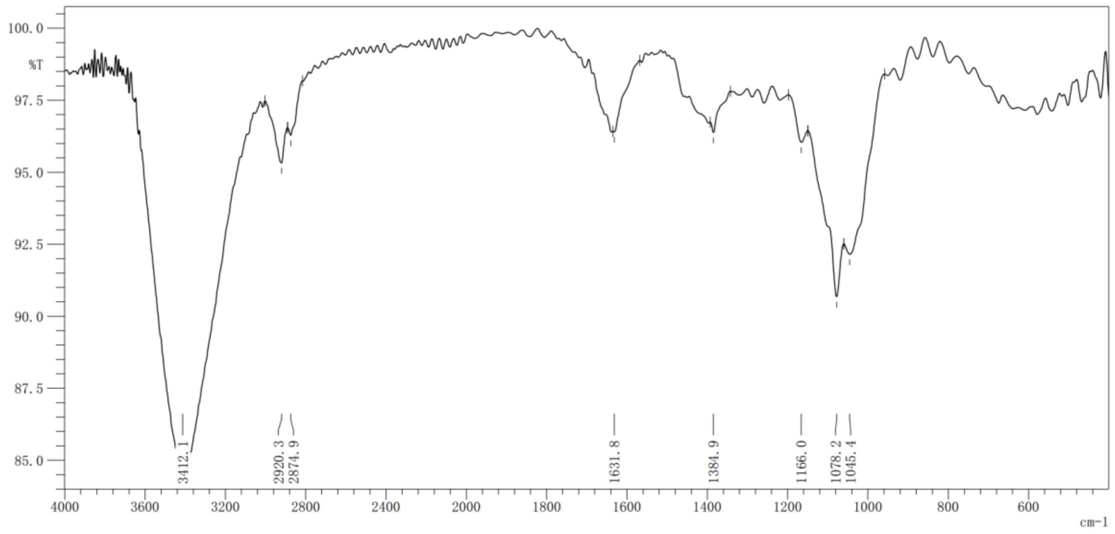

Figure S32. IR spectrum of compound **4**.

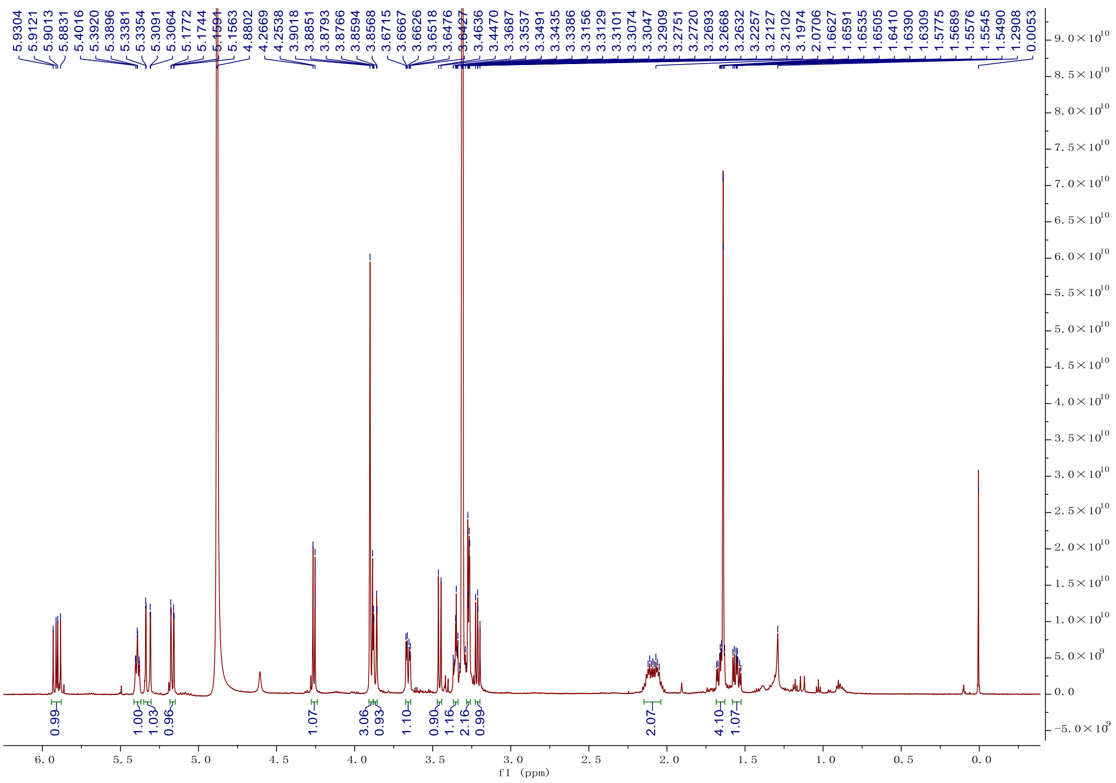

Figure S33. ^{1}H-NMR (600MHz, CD_3OD) spectrum of compound **4**.

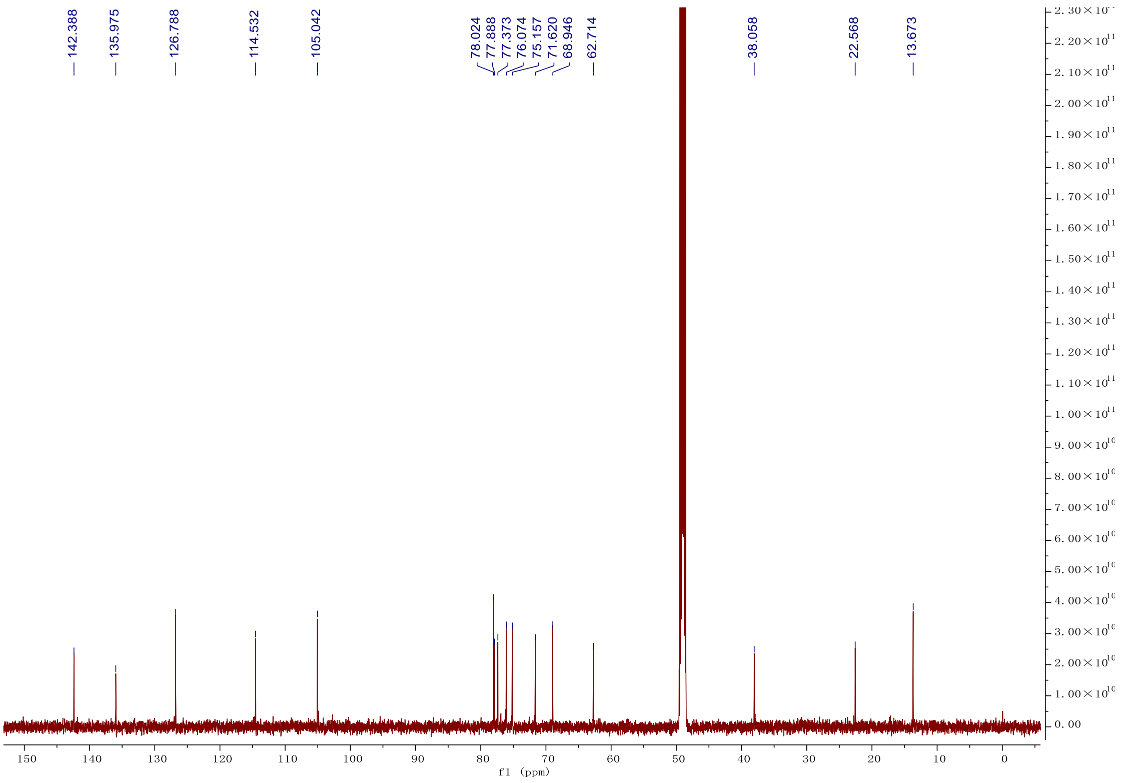

Figure S34. ^{13}C-NMR (150MHz, CD_3OD) spectrum of compound **4**.

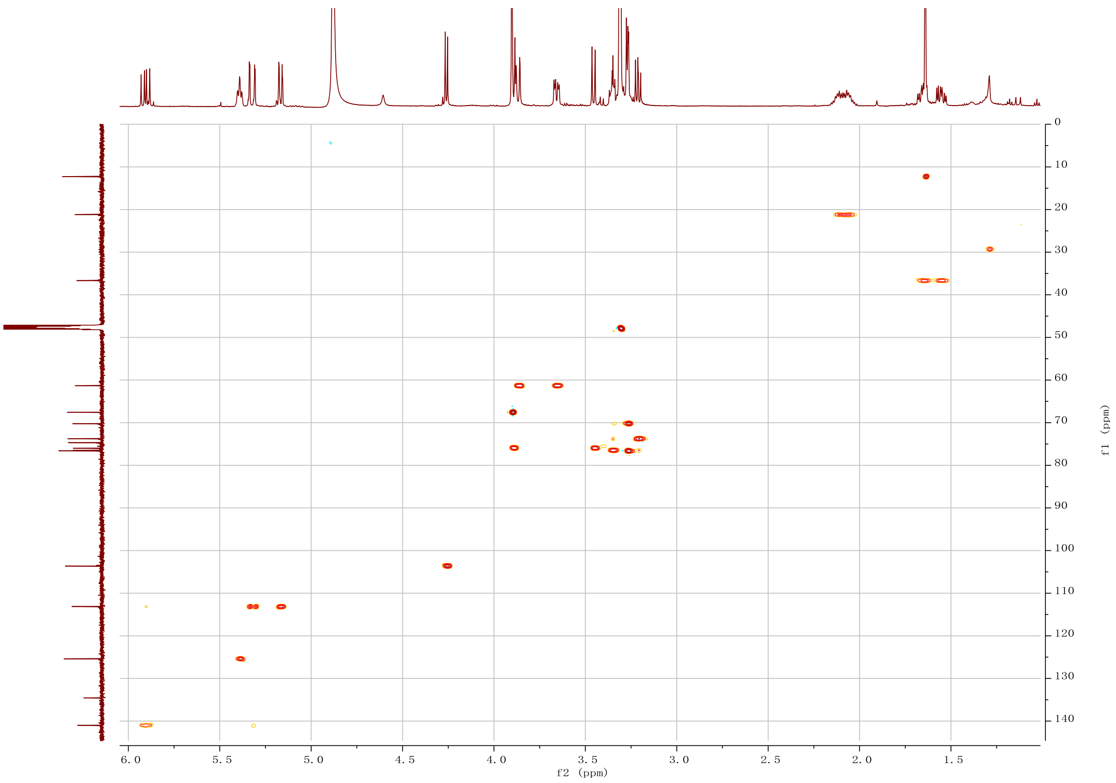

Figure S35. HMQC spectrum of compound **4**.

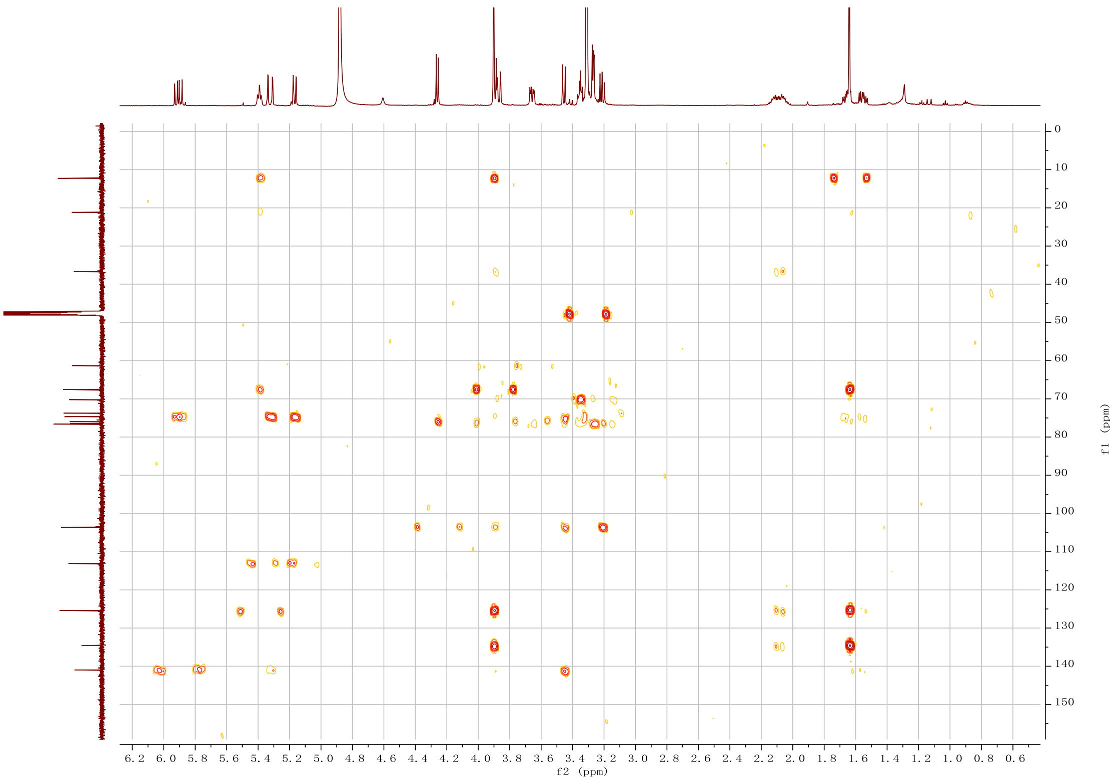

Figure S36. HMBC spectrum of compound **4**.

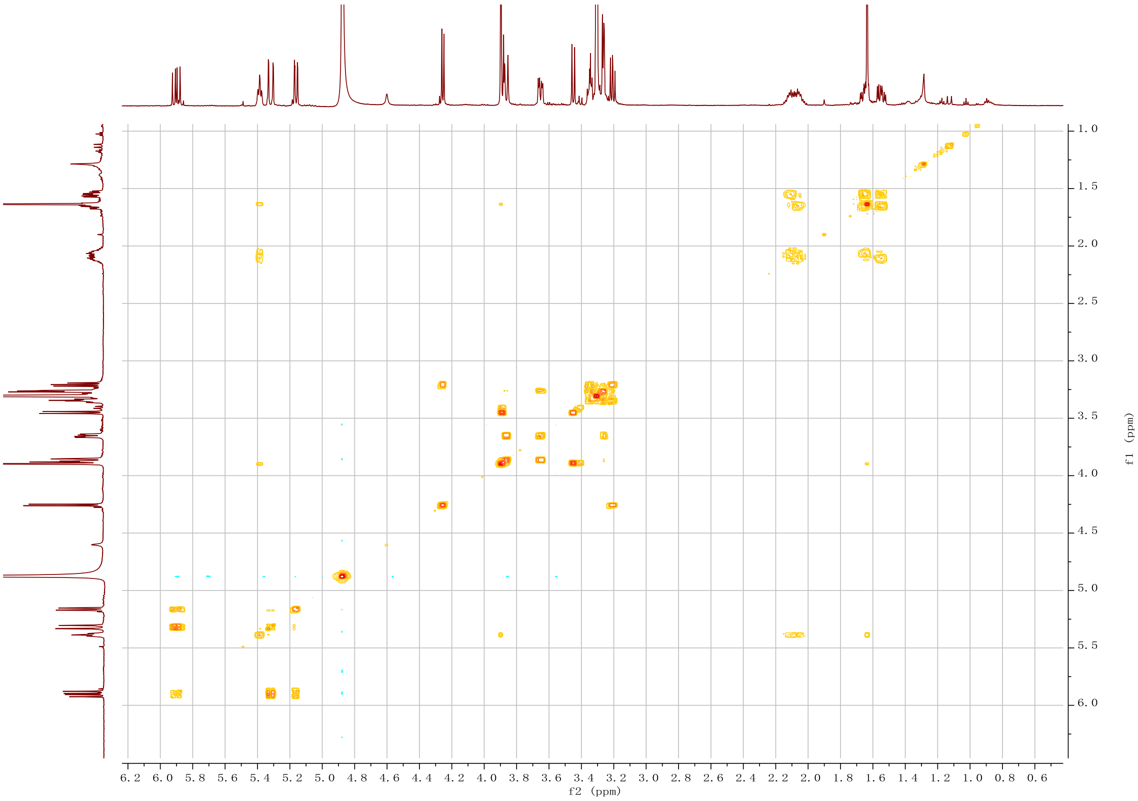

Figure S37. ^{1}H-^{1}H COSY spectrum of compound **4**.

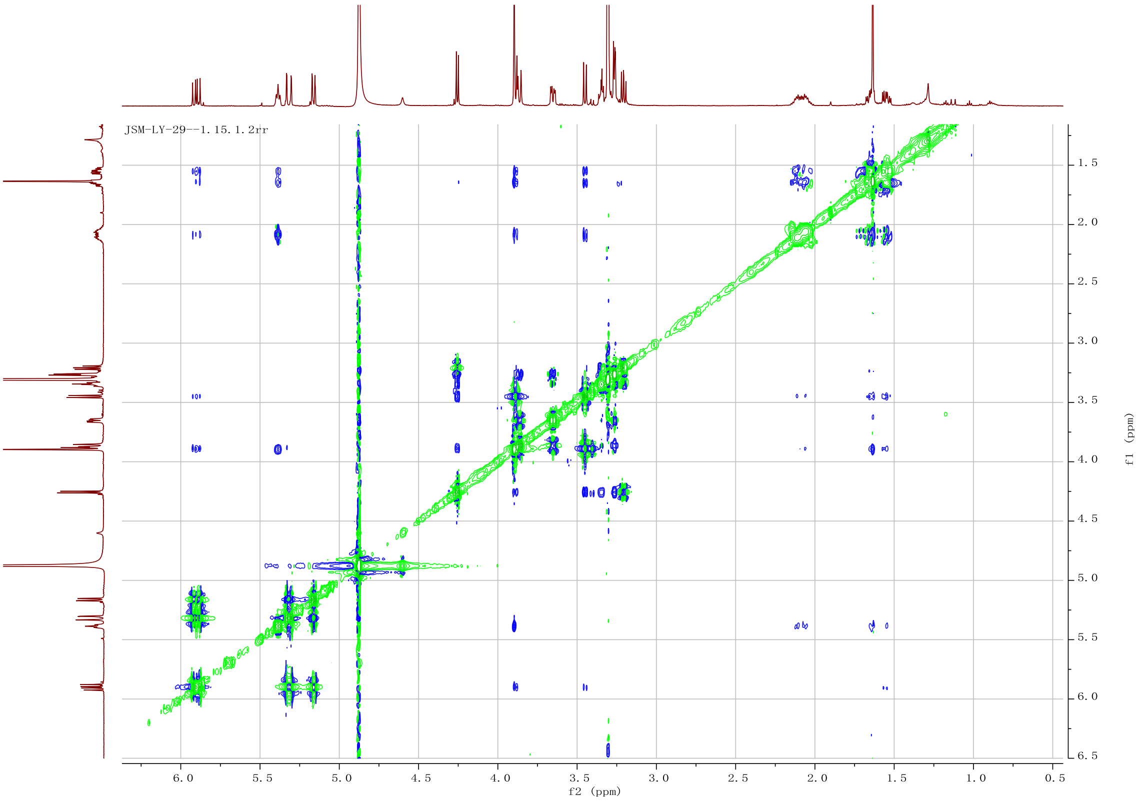

Figure S38. NOESY spectrum of compound **4**.

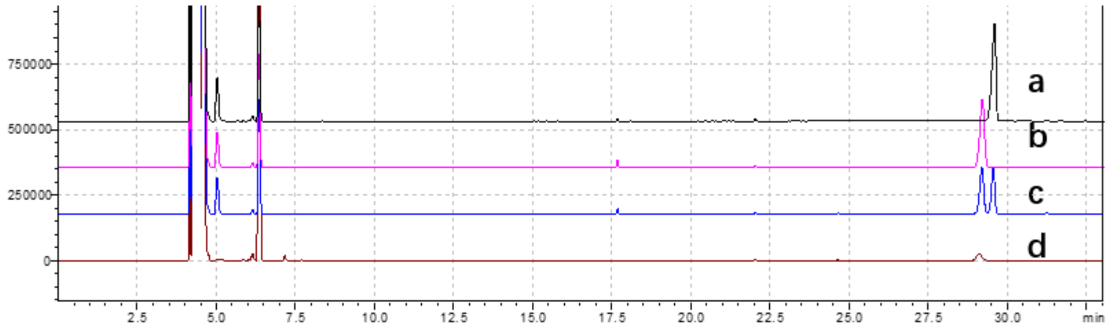

Figure S39. GC spectrum of saccharides hydrolysis and derivatization of compound **4.**

(a. derivatives of L glucose, t_R L-glucose derivative 29.56 min; b. derivatives of standard D, t_R D-glucose derivative 29.20 min; c. derivatives of standard D and L glucose, d. acid hydrolysis derivative of compound **4**.)

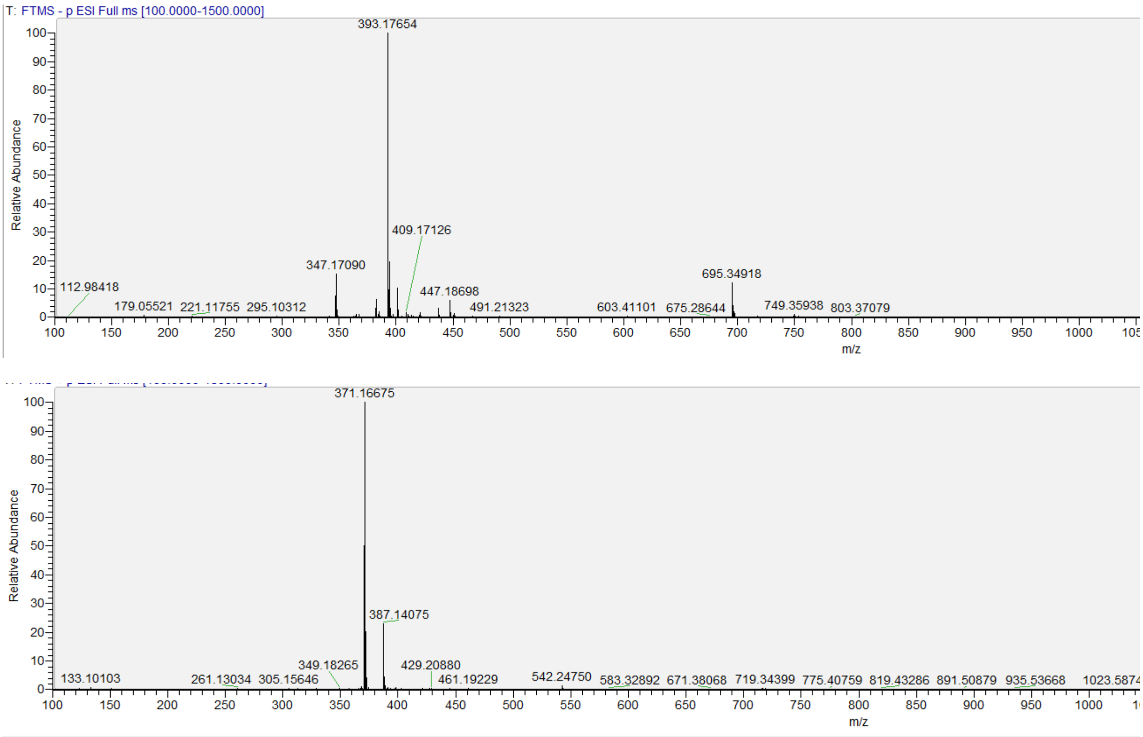

Figure S40. HR-ESI-MS spectrum of compound **5**.

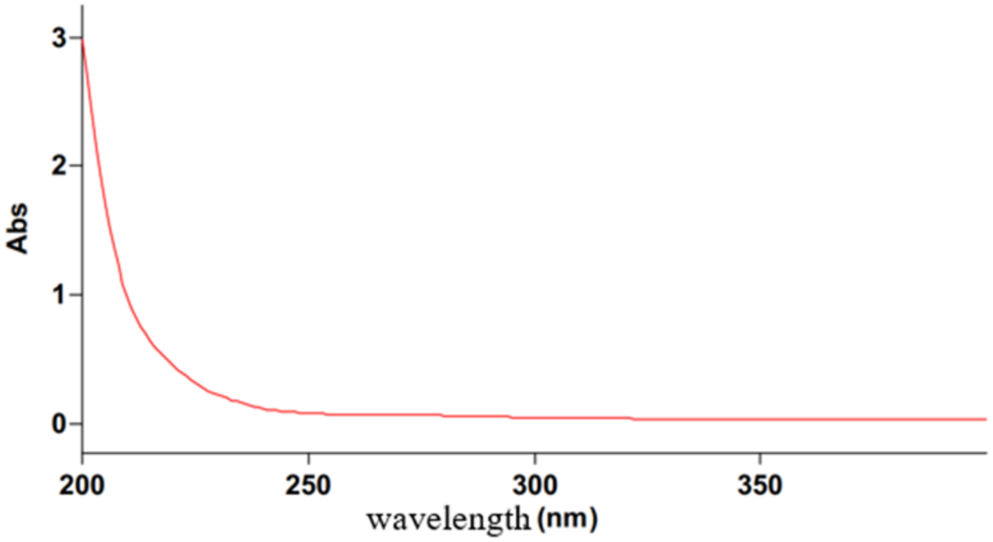

Figure S41. UV spectrum of compound **5**.

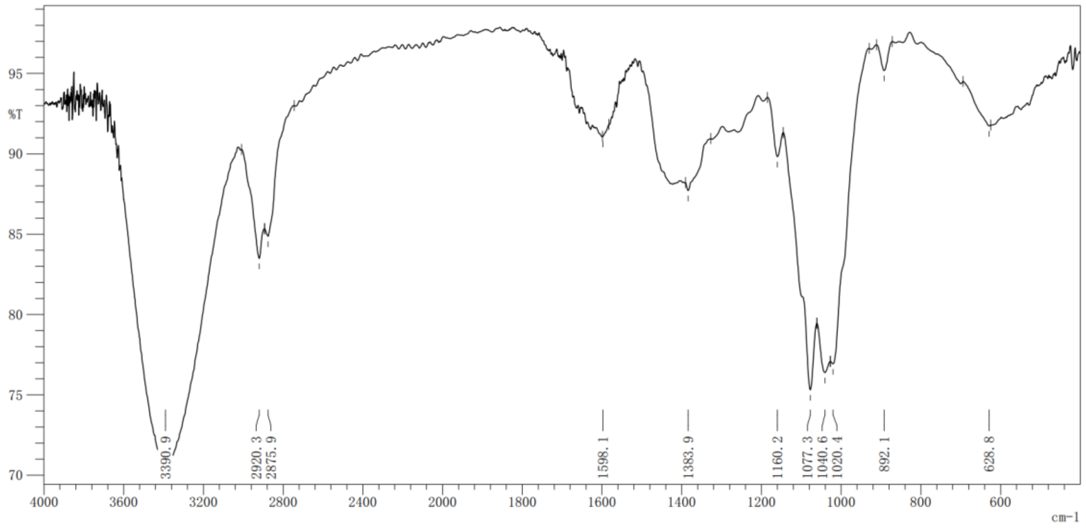

Figure S42. IR spectrum of compound **5**.

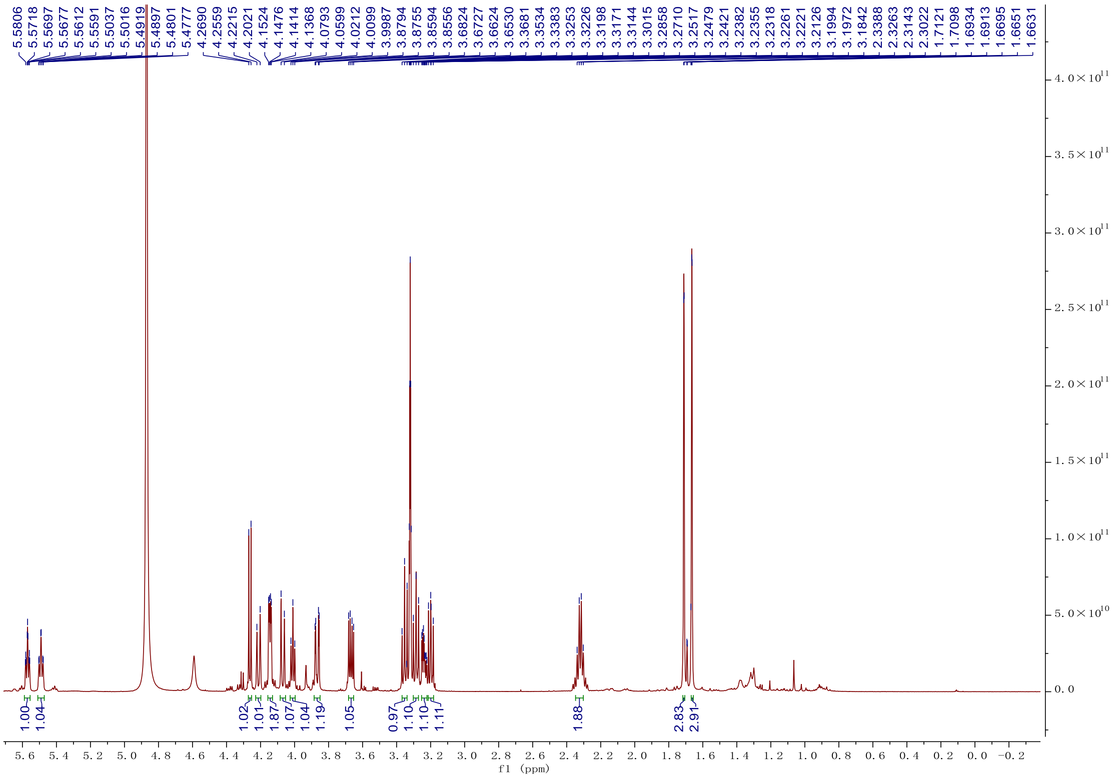

Figure S43. ^{1}H-NMR (600MHz, CD_3OD) spectrum of compound **5**.

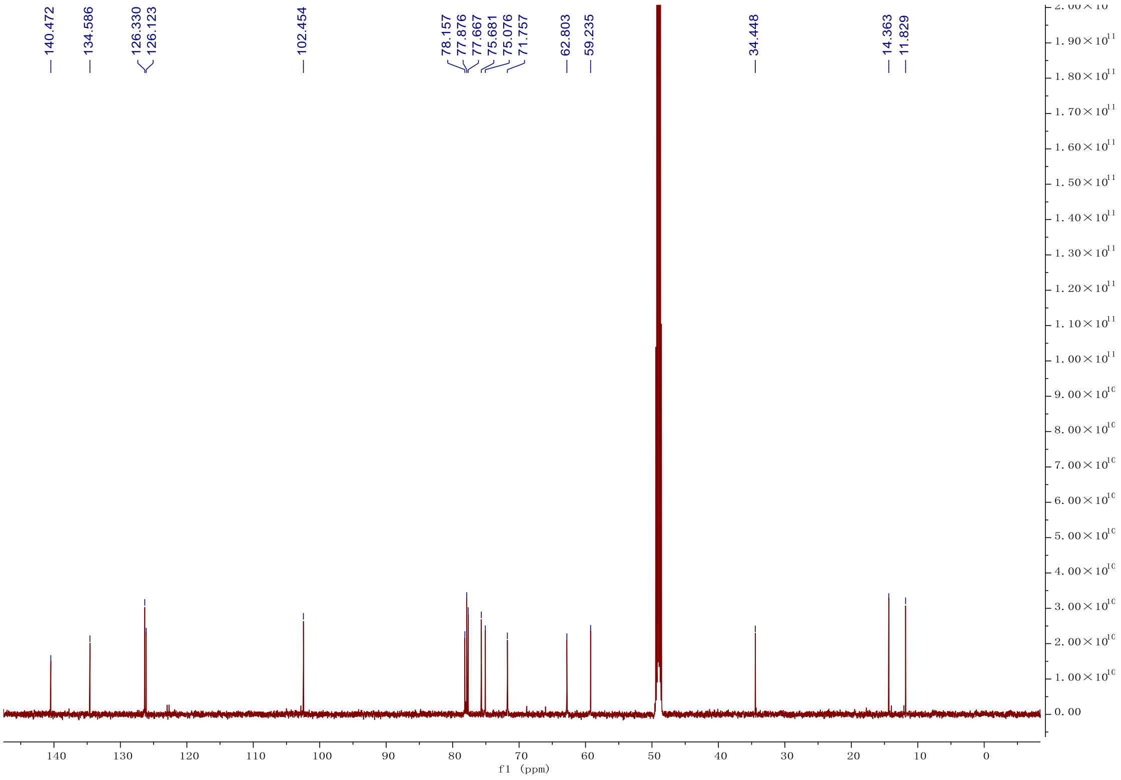

Figure S44. ^{13}C-NMR (150MHz, CD_3OD) spectrum of compound **5**.

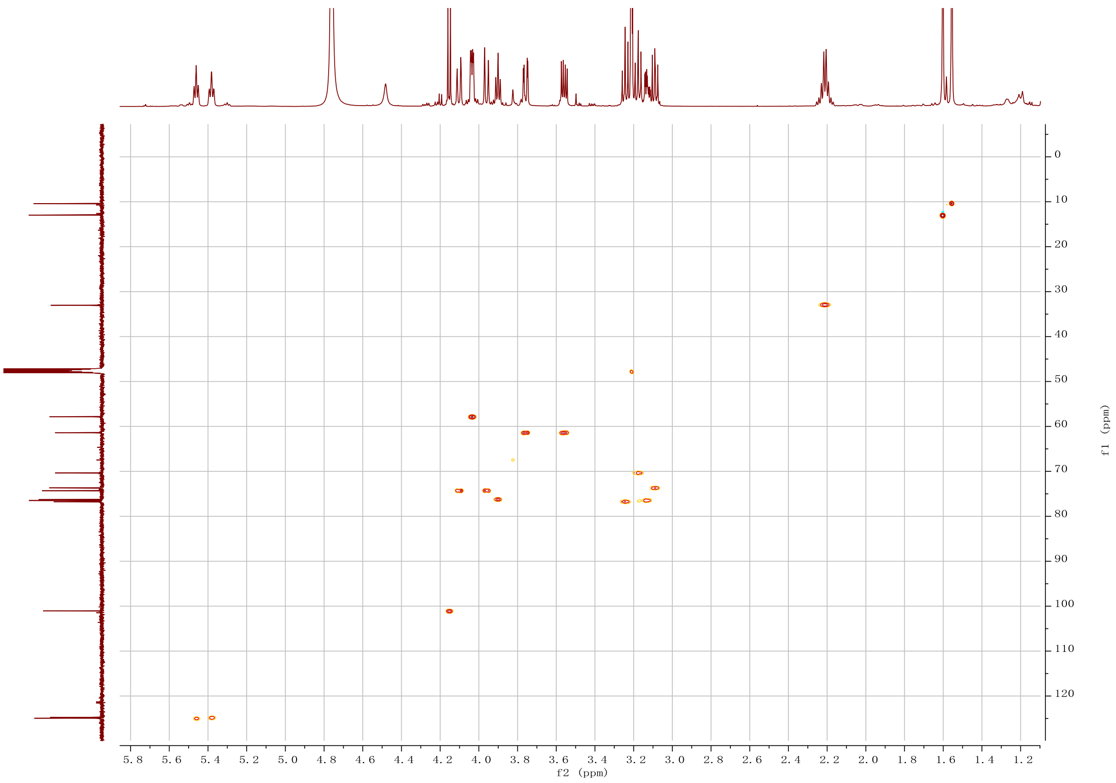

Figure S45. HMQC spectrum of compound **5**.

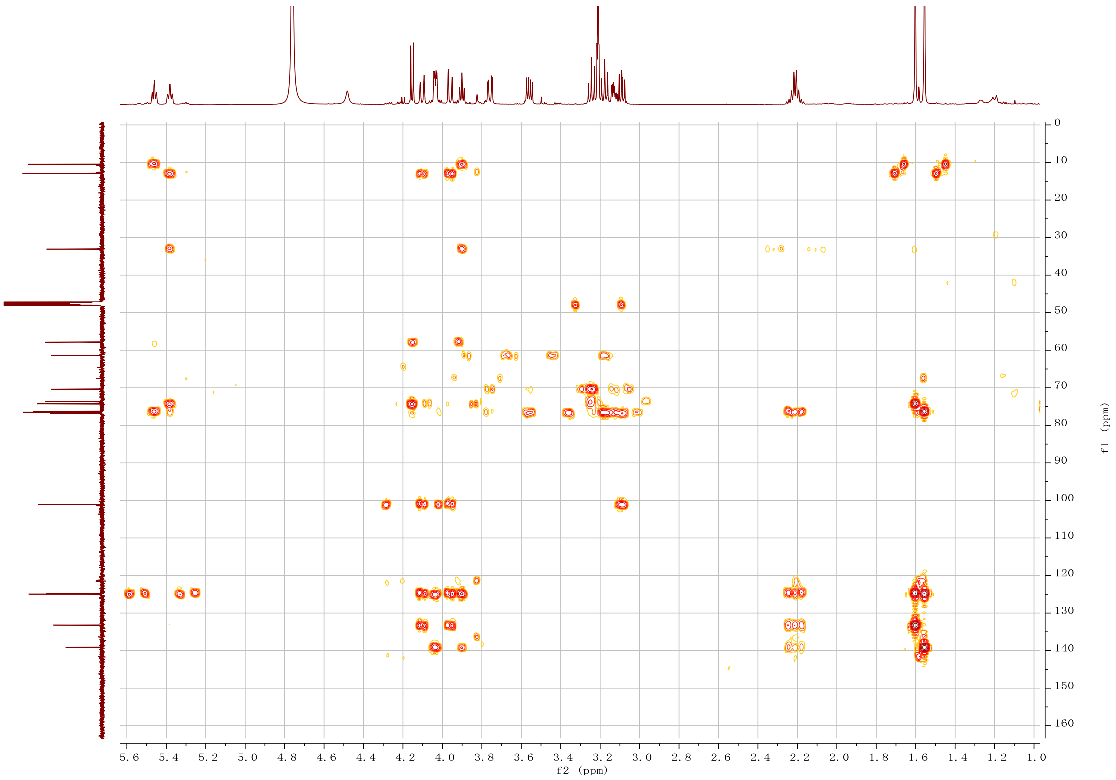

Figure S46. HMBC spectrum of compound **5**.

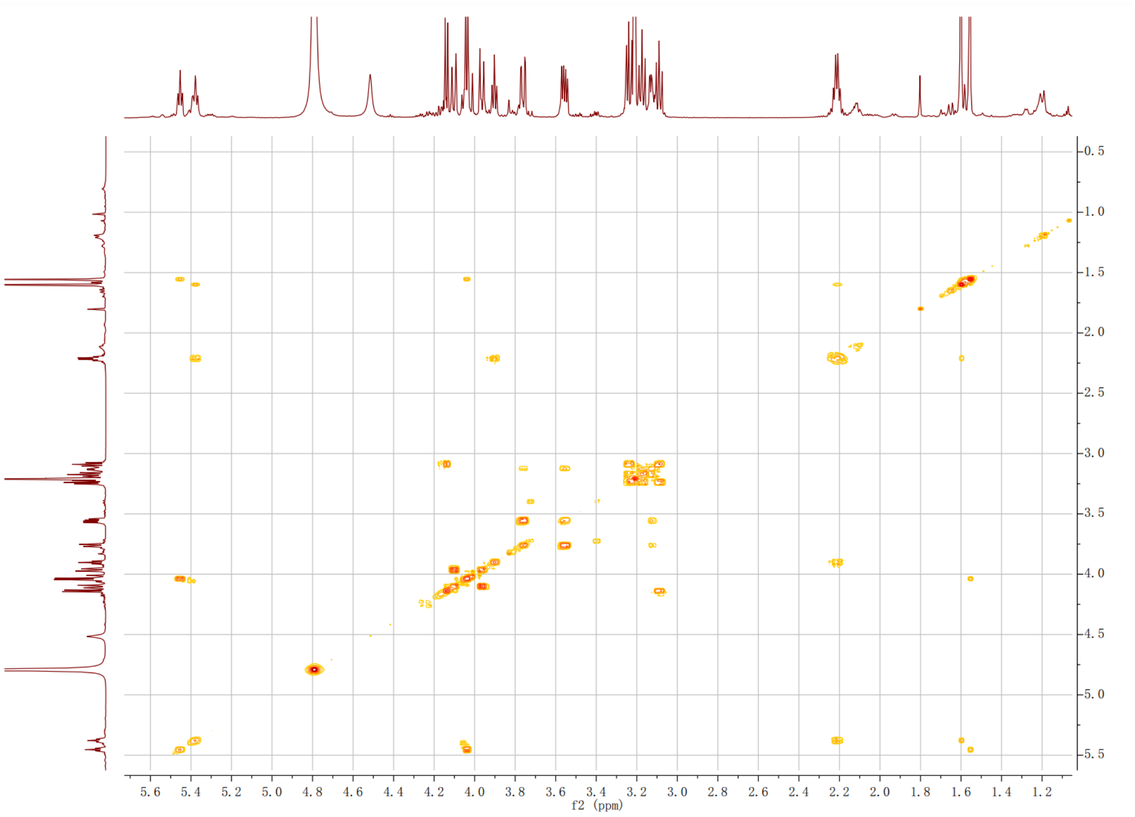

Figure S47. ^{1}H-^{1}H COSY spectrum of compound **5**.

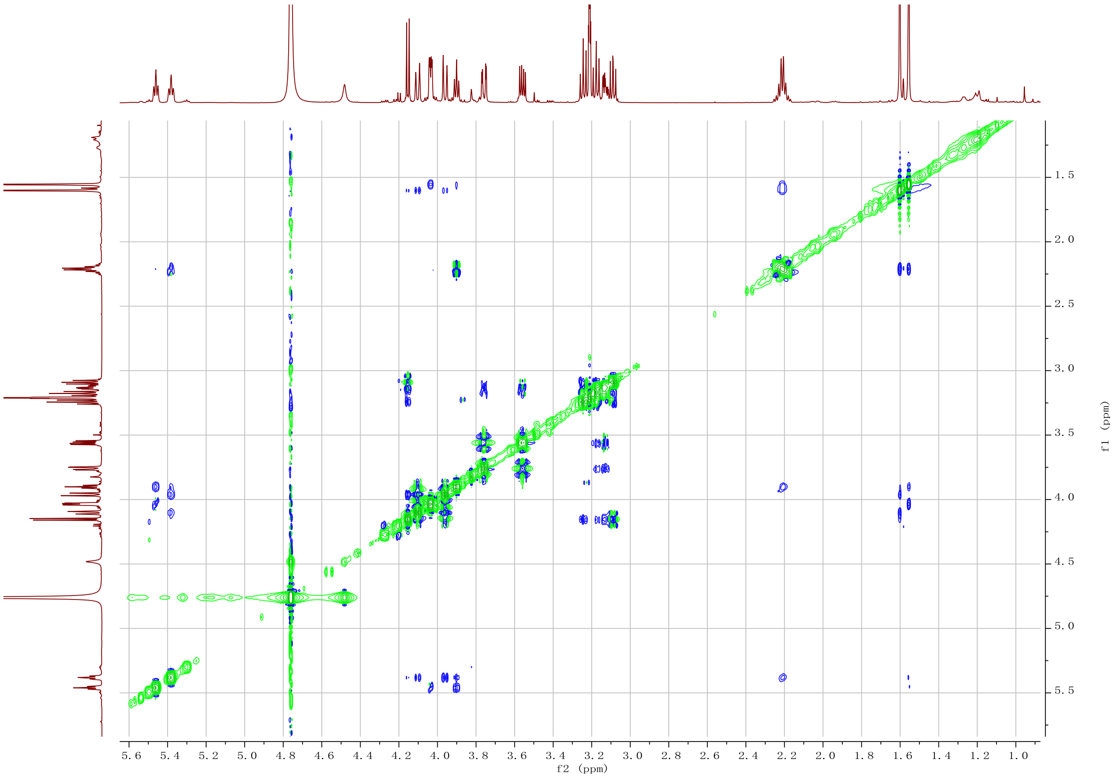

Figure S48. NOESY spectrum of compound **5**.

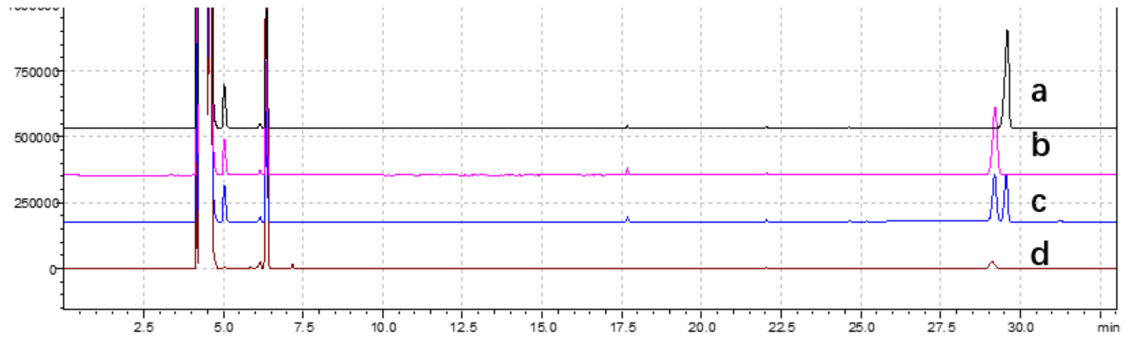

Figure S49. GC spectrum of saccharides hydrolysis and derivatization of compound **5.**

(a. derivatives of L glucose, t_R L-glucose derivative 29.56 min; b. derivatives of standard D, t_R D-glucose derivative 29.20 min; c. derivatives of standard D and L glucose, d. acid hydrolysis derivative of compound **5**.)
